# The Epigenetic Regulator G9a Mediates Tolerance to RNA Virus Infection in *Drosophila*

**DOI:** 10.1371/journal.ppat.1004692

**Published:** 2015-04-16

**Authors:** Sarah H. Merkling, Alfred W. Bronkhorst, Jamie M. Kramer, Gijs J. Overheul, Annette Schenck, Ronald P. Van Rij

**Affiliations:** 1 Department of Medical Microbiology, Radboud Institute for Molecular Life Sciences, Radboud University Medical Center, Nijmegen, The Netherlands; 2 Department of Human Genetics, Donders Institute for Brain, Cognition and Behaviour, Radboud University Medical Center, Nijmegen, The Netherlands; University of Edinburgh, UNITED KINGDOM

## Abstract

Little is known about the tolerance mechanisms that reduce the negative effects of microbial infection on host fitness. Here, we demonstrate that the histone H3 lysine 9 methyltransferase *G9a* regulates tolerance to virus infection by shaping the response of the evolutionary conserved Jak-Stat pathway in *Drosophila*. *G9a*-deficient mutants are more sensitive to RNA virus infection and succumb faster to infection than wild-type controls, which was associated with strongly increased Jak-Stat dependent responses, but not with major differences in viral load. Genetic experiments indicate that hyperactivated Jak-Stat responses are associated with early lethality in virus-infected flies. Our results identify an essential epigenetic mechanism underlying tolerance to virus infection.

## Author Summary

Multicellular organisms deploy various strategies to fight microbial infections. Invading pathogens may be eradicated directly by antimicrobial effectors of the immune system. Another strategy consists of increasing the tolerance of the host to infection, for example, by limiting the adverse effects of the immune response. The molecular mechanisms underlying this novel concept remain largely uncharacterized. Here, we demonstrate that the epigenetic regulator *G9a* mediates tolerance to virus infection in *Drosophila*. We found that *G9a*-deficient flies succumb faster than control flies to infection with RNA viruses, but that the viral burden did not significantly differ. Unexpectedly, mutant flies express higher levels of genes that are regulated by the Jak-Stat signaling pathway, which in other studies was found to be important for antiviral defense. Exploiting the genetic toolbox in *Drosophila*, we demonstrate that Jak-Stat hyperactivation induces early mortality after virus infection. Precise control of immune pathways is essential to ensure efficient immunity, while preventing damage due to excessive immune responses. Our results indicate that *G9a*, an epigenetic modifier, dampens Jak-Stat responses to prevent immunopathology. Therefore, we propose epigenetic regulation of immunity as a new paradigm for disease tolerance.

## Introduction

Efficient immunity against pathogens requires the coordinated activation and repression of genes within multiple signaling networks. Insufficient immune activation results in high microbial burden, severe pathogenesis, and high mortality from infection; overly strong immune responses may lead to tissue damage, immunopathology, and auto-inflammatory diseases. The inevitable tradeoff between immunity and immunopathology necessitates tightly regulated induction and resolution of immune responses. This is achieved by negative regulatory circuits within and among immune signaling cascades and by complex cellular and molecular programs that terminate inflammation [1,2].

It was recently proposed that host defense depends on a combination of resistance mechanisms, which lower or eliminate pathogen burden, and tolerance mechanisms [3,4]. Tolerance reduces the negative effects of an infection on host fitness, which could be either direct damage inflicted by the pathogen itself or adverse effects of the immune response on host tissues. Little is known about the molecular basis for tolerance, but it likely involves regulatory mechanisms that control the magnitude of the immune response [3,4].

The fruit fly *Drosophila melanogaster* is a powerful model to genetically and functionally dissect innate immunity. Past studies found that the evolutionarily conserved NF-κB pathways Toll and Immune Deficiency (Imd) mediate the humoral response against bacteria and fungi [5]. Defense against viruses, in contrast, requires the constitutively expressed RNA interference (RNAi) pathway [6]. In addition, the RNA viruses Drosophila C virus (DCV), Cricket paralysis virus (CrPV), and Drosophila X virus (DXV) activate the Janus Kinase-Signal transducers and activators of transcription (Jak-Stat) pathway that orchestrates a transcriptional response to fight the infection [7,8].

The evolutionarily conserved Jak-Stat pathway controls important developmental and homeostatic processes, including hematopoiesis and immunity [9,10]. Deficiencies in Jak-Stat pathway genes cause serious immune disorders and increase susceptibility to infections [11–13], whereas hyperactivated Jak-Stat responses are associated with autoimmune diseases and carcinogenesis in humans [13,14]. Also in insects, the Jak-Stat pathway needs to be tightly controlled. The Jak-Stat pathway is required for efficient antiviral immunity in fruit flies and *Aedes aegypti* mosquitoes, [15]. For example, loss-of-function fly mutants for the Jak kinase *hopscotch* (*hop*) support high levels of virus replication and show increased mortality rates upon infection with the RNA viruses DCV and CrPV [7,8]. Yet, hyperactivation of the Jak-Stat pathway in *Drosophila* can have serious consequences, such as the formation of lethal hematopoietic melanotic tumors in *hop* gain-of-function mutants [16].

Spatiotemporal regulation of immune responses occurs via a variety of mechanisms. At the transcriptional level, chromatin structure is a major determinant of gene expression. Histone-modifying enzymes deposit covalent modifications on specific amino acid residues of histone tails that alter the structure of chromatin and its accessibility to the transcriptional machinery. Histone H3 lysine 9 dimethylation (H3K9me2) is commonly regarded as a marker for heterochromatic genomic regions and transcriptional repression. Yet, *G9a*, one of the three H3K9 methyltransferases in *Drosophila*, mediates H3K9 dimethylation *in vivo*, but is associated with euchromatic regions [17]. Loss of *G9a* does not affect heterochromatin formation or global heterochromatic H3K9me2 levels in flies and mice [18,19], but *G9a* fly mutants show loss of H3K9 dimethylation at about 5% of the euchromatic genome [20]. Moreover, H3K9me2 can be associated with actively transcribed genes [21] and its presence does not globally correlate well with gene repression, unlike other repressive marks such as H3K27me2 and H3K27me3 [20,22]. These observations suggest that H3K9me2 is not solely associated with stably repressed genes, and that *G9a* might regulate defined sets of euchromatic genes.

A previous study revealed that *G9a* controls genes that are involved in processes that require tight and dynamic regulation and high transcriptional plasticity, including neuronal processes, stress responses, and immunity [20]. These observations prompted us to study the role of *G9a* in antiviral defense. Here, we report that *G9a* mutant flies are hypersensitive to RNA virus infection and that their inducible immune responses are highly dysregulated. We show that *G9a* and the Jak-Stat pathway epigenetically and genetically interact to modulate immune defense. Genetic hyperactivation of Jak-Stat signaling causes early lethality after viral infection, thus phenocopying loss of *G9a*. Together, our results uncover an epigenetic mechanism for tolerance that shapes Jak-Stat pathway activity in response to virus infection.

## Results

### Reduced survival of *G9a* mutant flies after RNA virus infection

**Fig 1:**
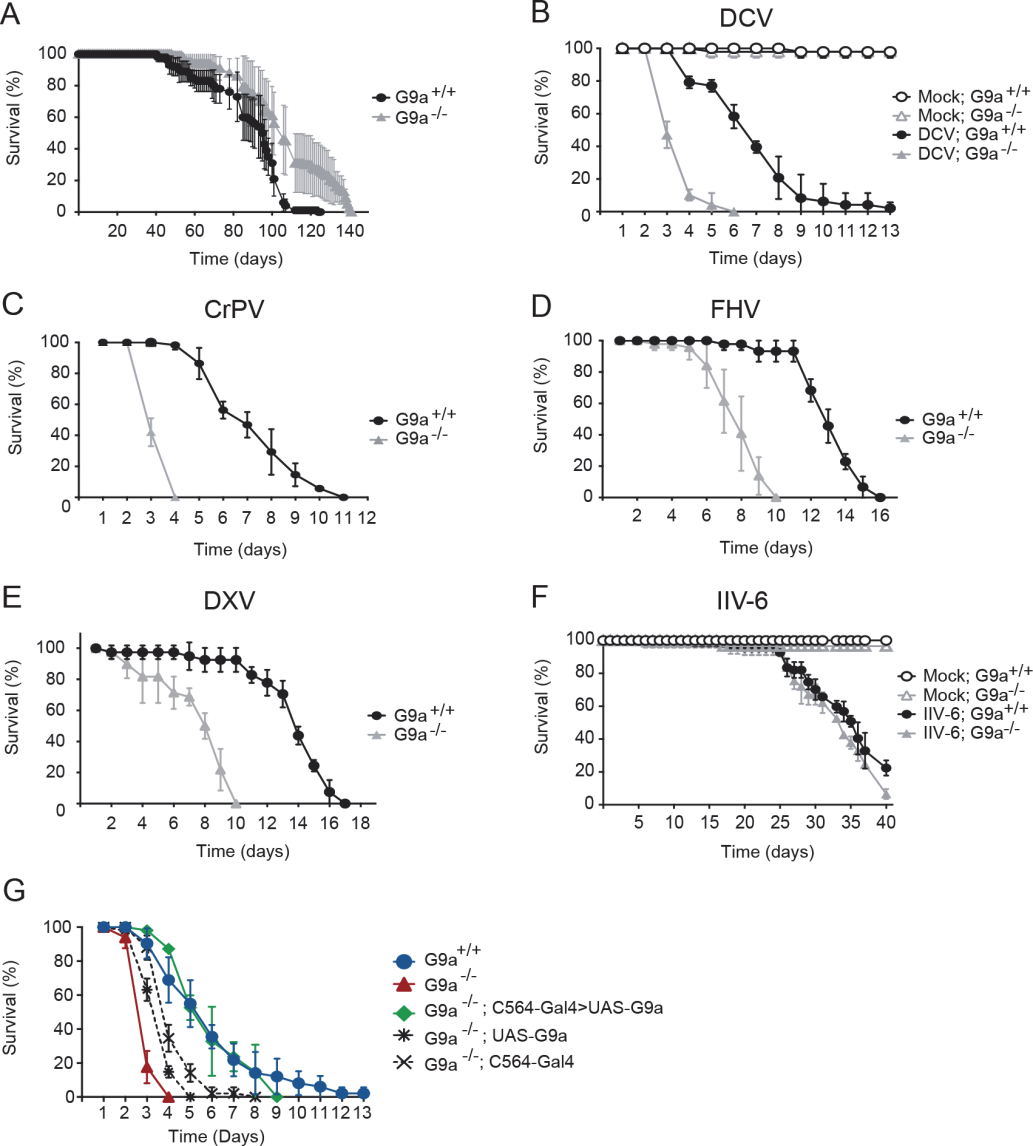
*G9a* mutants have a normal life span but are hypersensitive to RNA virus infection. (**A**) Life span of non-infected female wild-type (*G9a* ^+/+^) or *G9a* mutant (*G9a* ^-/-^) flies at 20°C. (**B-F**) Survival of wild-type or *G9a* mutants infected with (**B**) DCV, (**D**) CrPV, (**D**) FHV, (**E**) DXV, (**F**) IIV-6, and Tris buffer as a control (mock). (**G**) Survival of wild-type or *G9a* mutant flies expressing a *G9a* transgene in the fat body using the UAS/Gal4 system upon DCV infection (1,000 TCID_50_ units). The fat body-specific C564 driver line (*C564-Gal4*) was used to drive expression of the transcription factor Gal4, which binds to the Upstream Activating Sequence to induce expression of a *G9a* transgene (*UAS-G9a*). Flies expressing only the *C564-Gal4* driver or the *UAS-G9a* responder construct were included as controls. Mock infections were performed in all experiments (**B**-**G**), and no difference in survival was observed between wild-type or *G9a* mutant flies, as shown in panel **B** and **F**. All survival data are available in S1 Dataset. Data represent means and s.d. of five (**A**) or three (**B-G**) biological replicates of at least 15 female flies (**A**-**F**), or 15 male flies (**G**) per replicate for each genotype. Data are from one experiment representative of at least 3 (**B**,**C**,**F**,**G**), or 2 (**D**,**E**) independent experiments.

To investigate the role of *G9a* in *Drosophila* antiviral defense, we used the loss-of-function allele *G9a* ^DD2^ and its wild-type genetic background control (hereafter referred to as *G9a* ^-/-^ and *G9a* ^+/+^)[20]. Since the H3K9me2 mark is essential for the establishment of heterochromatin and proper gene regulation, we first assessed the overall fitness of *G9a-*deficient flies. *G9a* mutants were viable, fertile, and showed no obvious defects in development, confirming previous observations [20,23]. Moreover, the average life span of *G9a* ^-/-^ flies was slightly longer than that of wild-type controls (mean survival = 105.8 days and 87,8 days, respectively; *P* < 0.001) (Fig 1A).

We challenged wild-type and mutant flies with DCV, a positive-sense RNA virus from the *Dicistroviridae* family, by intra-thoraxic injection. *G9a* mutants were more sensitive to infection than their wild-type controls, with a mean survival of 3.6 and 6.9 days for *G9a* ^-/-^ and *G9a* ^+/+^ female flies, respectively (*P* < 0.001; Fig 1B). Male *G9a-*deficient flies were also more sensitive to DCV infection than control flies, indicating that hypersensitivity to virus infection was not sex-dependent (S1A Fig).

To analyze whether *G9a* ^-/-^ flies were also more sensitive to other virus infections, we challenged flies with a panel of viruses with different genome organization and genetic makeup. Upon challenge with another Dicistrovirus, Cricket paralysis virus (CrPV), the mean survival of *G9a* mutants was 3.4 days, compared to 7.3 days for wild-type flies (*P* < 0.001; Fig 1C). Similarly, when infected with Flock House Virus (FHV), a positive-sense virus of the *Nodaviridae* family, *G9a* mutants succumbed faster than wild-type flies to infection (mean survival = 7.9 days and 13.1 days, respectively; *P* < 0.001; Fig 1D). Also upon challenge with the dsRNA virus Drosophila X Virus (DXV, member of the *Birnaviridae*), *G9a* mutant flies displayed higher lethality rates compared to wild-type controls (mean survival 7.6 days and 13.6 days, respectively, *P* < 0.001; Fig 1E). To analyze whether *G9a* mutants are also more sensitive to DNA virus infection, we challenged flies with Invertebrate iridescent virus 6 (IIV-6). As we observed before, IIV-6 infected wild-type flies survived for prolonged periods of time and mortality only became apparent in the later stages of the infection (>25 days post infection) [24]. In contrast to their hypersensitivity to RNA virus infection, survival rates of *G9a* mutants after IIV-6 infection were similar to wild-type levels (mean survival = 33.6 days and 34.4 days, respectively; *P* = 0.2; Fig 1F). Of note, mock infection with Tris buffer did not affect the survival rate of *G9a* mutants for up to 40 days (Fig 1F). Flies carrying another loss-of-function allele, *G9a* ^DD3^, exhibited the same phenotype and succumbed more rapidly than their wild-type controls to DCV, but not to IIV-6 infection (S1B–S1D Fig). As *G9a* mutants displayed increased sensitivity against all RNA viruses tested, we used the model RNA virus DCV for follow-up studies.

### Fat body specific expression of *G9a* rescues hypersensitivity to virus infection

To confirm the role of *G9a* in antiviral defense, we performed genetic rescue experiments by expression of a *G9a* transgene in the mutant background using the UAS/Gal4 system. We were unable to recover adult flies expressing the *G9a* transgene under control of the drivers *actin-Gal4*, *daughterless-Gal4* and *tubulin-Gal4*, suggesting that ubiquitous overexpression of *G9a* is detrimental to fly development. The fat body, an organ that is involved in metabolism and immunity [5], is a major target organ of DCV [25]. We therefore used a fat body driver (*C564-Gal4*) to induce tissue-specific expression of the *G9a* transgene. Early lethality of infected *G9a* mutants (mean survival = 3.1 days) was rescued to control levels by fat body-specific *G9a* expression in the *G9a*-deficient background (mean survival = 5.6 days, compared to 6.3 days for *G9a* ^+/+^; *P* = 0.482; Fig 1G). Survival of genetic control flies that only express the C564-Gal4 driver or the UAS-responder in the *G9a* ^-/-^ background remained significantly different from wild-type flies (mean survival = 4.1 and 3.8 days, respectively, P < 0.001 for both), indicating that the observed rescue was dependent on functional expression of the *G9a* transgene (Fig 1G). The rescue was tissue specific, since expression of *G9a* in other tissues, such as hemocytes (using the *hemolectin-Gal4* driver), or glia (using the *repo-Gal4* driver) did not rescue the phenotype of *G9a* mutants (S2A and S2B Fig). These experiments indicate that *G9a* is required specifically in the fat body during virus infection. Moreover, these experiments genetically segregate the role of *G9a* in antiviral defense from its function in other organs [20].

### Reduced tolerance of *G9a* mutants to RNA virus infection

**Fig 2:**
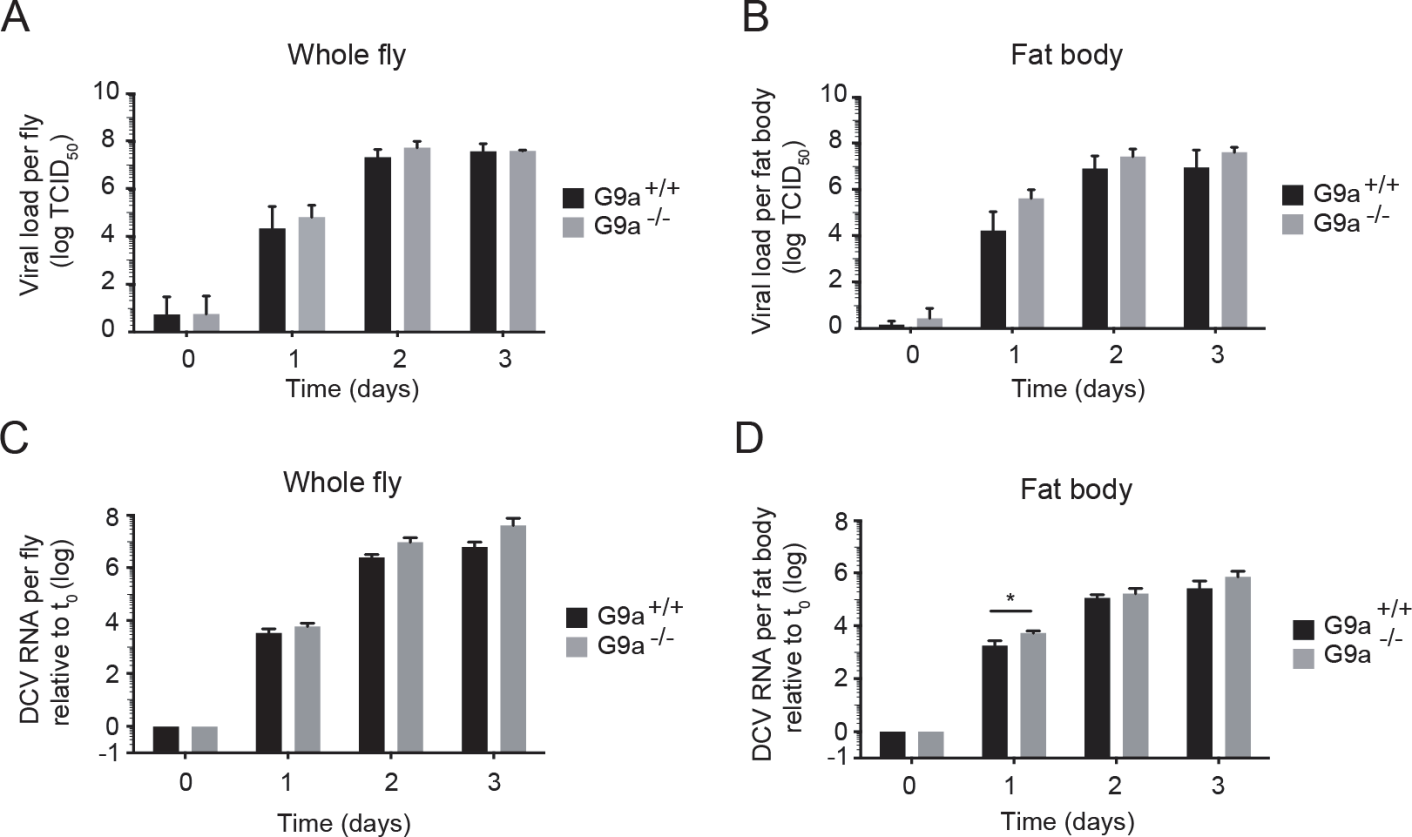
Loss of *G9a* does not affect viral loads upon DCV infection. (**A,B**) Wild-type or *G9a* mutant flies were inoculated with DCV and viral titers were determined over time in (**A**) whole flies, and (**B**) dissected fat bodies. Data represent means and s.d of three independent experiments. Each experiment contained three biological replicates of 5 female flies (**A**), or 10 fat bodies (**B**) per replicate for each genotype. (**C**,**D**) DCV RNA levels over the course of 3 days post-infection analyzed by RT-qPCR in (**C**) whole flies or (**D**) fat bodies of wild-type and *G9a* mutant flies. DCV RNA levels were normalized to transcript levels of the housekeeping gene *Ribosomal Protein 49* and are calculated relative to the viral RNA levels in flies harvested immediately after inoculation (t_0_). Data represent means and s.d. of three biological replicates of 5 female flies (**C**) or 10 fat bodies (**D**) per replicate for each genotype. Data in panel **C** and **D** are from one experiment representative of 2 independent experiments. *P < 0.05 (Student’s t-test).

To analyze whether the reduced survival of *G9a* mutants is due to a defect in resistance or to reduced tolerance to infection, we analyzed viral load over time. No differences in infectious viral titers were observed between *G9a* ^-/-^ and *G9a* ^+/+^ flies during the first 3 days post-infection (dpi) (Fig 2A). Since *G9a* was specifically required in the fat body during DCV infection (Fig 1G), we analyzed viral titers in dissected fat bodies of virus-challenged flies. Virus titers in *G9a* ^-/-^ flies were slightly higher than in wild-type flies, but no significant difference was observed at any time point (Fig 2B). To confirm these data, we measured viral RNA levels in whole flies and fat bodies by quantitative RT-PCR (RT-qPCR). Consistent with the results from the titration, we did not detect significant differences in DCV RNA levels in wild-type and mutant flies over three days post-infection. In the fat body, we observed a modest 3-fold increase in viral RNA at 1 dpi (*P* = 0.014), but not at the other time points (Fig 2C and 2D).

Together, our results demonstrate that *G9a* mutants are more sensitive to DCV infection, but that this is not associated with a major and generalized increase in viral titers. Moreover, the modest increase in viral load at 1 dpi in the fat body seems insufficient to explain the strongly reduced survival upon virus infection. We conclude that *G9a* mutant flies exhibit defects in tolerance to RNA virus infection.

### The antiviral RNAi pathway is functional in *G9a* mutants

RNA interference (RNAi) is a major antiviral pathway in *Drosophila* [6]. Given the hypersensitivity of *G9a* ^-/-^ flies to virus infection, we analyzed whether this pathway is functional in mutant flies. To this end, we first monitored RNAi activity using an *in vivo* sensor assay, in which the inhibitor of apoptosis *thread* (*th*) is silenced by expression of an RNAi-inducing hairpin RNA (*th* ^RNAi^) [26,27]. Expression of *th* ^RNAi^ using the eye-specific driver (*GMR-Gal4*) leads to severe apoptosis in the developing eye. Consequently, adult *th* ^RNAi^ flies display a reduced eye size, roughening of the eye surface, and loss of pigmentation (S3A Fig). This phenotype is fully dependent on the RNAi pathway, since the phenotype is lost in mutants lacking the central catalytic component of the pathway, Argonaute 2 (*AGO2)* [26,27]. We expressed the *th* ^RNAi^ hairpin in the eye of *G9a* ^-/-^ and *G9a* ^+/+^ flies and analyzed the phenotype. Both in *G9a* ^-/-^ and *G9a* ^+/+^ flies, expression of *th* ^RNAi^ resulted in strong RNAi-induced eye phenotypes (S3A Fig). These results suggest that *G9a* ^-/-^ mutant flies have no major defect in RNAi.

To further evaluate the efficiency of the RNAi response of *G9a* mutants, we adapted a luciferase-based RNAi sensor assay that we routinely use in *Drosophila* S2 cells [27,28], to adult flies. Flies were subjected to *in vivo* transfection with firefly and *Renilla* luciferase reporter plasmids along with either firefly luciferase-specific dsRNA or control dsRNA, and three days later, efficiency of silencing was assessed in whole fly lysates. As controls, we included *Ago2* null mutants and their wild-type controls (*w* ^1118^). As expected, silencing was abolished in *Ago2* ^-/-^ flies, confirming that loss of FLuc expression was RNAi dependent (S3B Fig, left panel). Efficiency of silencing was similar in *G9a* ^-/-^ and *G9a* ^+/+^ flies (S3B Fig, right panel), indicating the RNAi pathway is fully proficient in *G9a* mutant flies.

### Hyperactivation of the Jak-Stat pathway in *G9a* mutants upon virus infection

Since the RNAi pathway was fully functional in *G9a* ^-/-^ flies, we next analyzed whether inducible immune responses were intact in these flies. Virus infection of *Drosophila* activates the Jak-Stat pathway to induce expression of downstream genes, such as *virus induced RNA-1* (*vir-1*) [7,8]. In addition, the NF-κB pathways Toll and IMD have been implicated in the response to virus infections in some studies [29–31]. We measured expression of *vir-1*, the stress-induced genes *Turandot A* and *M* (*TotA* and *TotM*), and the antimicrobial-like peptide *Listericin* as markers for Jak-Stat activation. To monitor activation of the Toll and IMD pathways, we measured expression of genes encoding the antimicrobial peptides *Drosomycin* (*Drs*), *Metchnikowin* (*Mtk*), *Diptericin* (*Dpt*). In addition, we measured expression of *Vago*, which is induced in DCV infection via an unknown signaling pathway [25].

**Fig 3:**
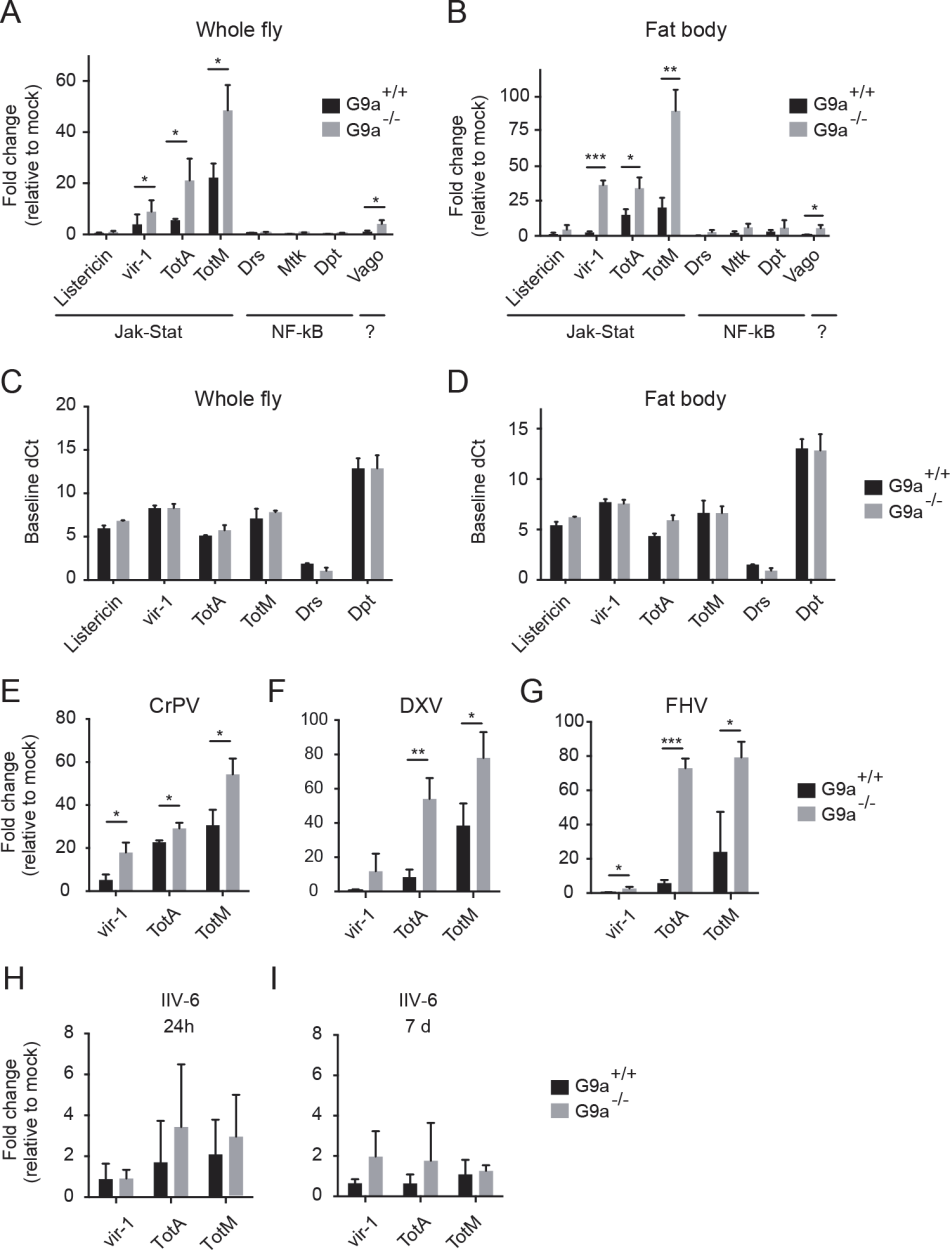
Hyperactivation of the Jak-Stat pathway by virus infection of *G9a* mutants. (**A**,**B**) Expression of inducible immune genes at 24 hours after DCV infection (TCID_50_ = 10,000) determined by RT-qPCR in (**A**) whole flies, and (**B**) fat bodies of wild-type or *G9a* mutant flies. Expression of the gene of interest was normalized to transcript levels of the housekeeping gene *Ribosomal Protein 49* and expressed as fold change relative to mock infection (Tris buffer). Data are means and s.d. of three independent pools of (**A**) 30 female flies and (**B**) 30 fat bodies for each genotype. (**C,D**) Basal expression levels of the indicated genes measured by RT-qPCR on 3 to 5-day-old unchallenged wild-type and *G9a* mutant female flies (**C**) or fat bodies **(D**). Basal expression levels are expressed as dCt values (difference between Ct of the gene of interest and the Ct of *Ribosomal Protein 49)*. (**E**-**I**) Expression of inducible Jak-Stat dependent immune genes at (**E**-**H**) 24 hpi or (**I**) 7 dpi with 10,000 TCID_50_ units of (**E**) CrPV, (**F**) DXV, (**G**) FHV or (**H**,**I**) 14,000 TCID_50_ units of IIV-6. Data are means and s.d. of three independent pools of at least 15 female flies (**C,E-I**) or at least 10 fat bodies (**D**) per genotype. Data are from one experiment representative of 3 (**A,B,E**), and 2 (**C**,**D**) independent experiments. *P < 0.05; ** *P* < 0.01; *** *P* < 0.001 (Student’s t-test).

We monitored expression of these genes by RT-qPCR at 24 hours after DCV infection (hpi) in whole flies (Fig 3A) and isolated fat bodies (Fig 3B). As observed before [7,8], DCV infection induced expression of the Jak-Stat dependent genes *vir-1*, *TotA*, and *TotM*, but not of NF-κB dependent *Drs*, *Mtk*, and *Dpt* genes. Strikingly, in *G9a* ^-/-^ flies we noted a much higher induction of Jak-Stat dependent genes than in wild-type flies, but no induction of NF-κB dependent genes (Fig 3A). In the fat body, even stronger overactivation of Jak-Stat dependent pathway genes was observed in *G9a* mutants (Fig 3B). However, basal expression levels of these genes did not differ between non-challenged *G9a* ^+/+^ and *G9a* ^-/-^ flies (Fig 3C and 3D), suggesting that *G9a* is not required for steady-state repression of these genes, but that it mitigates their inducibility in response to viral infection.

We also monitored expression of the Jak-Stat dependent genes upon infection with 3 other RNA viruses: CrPV, DXV and FHV (Fig 3E–3G). As observed upon DCV infection, a strong upregulation of *vir-1*, *TotA*, and *TotM* was found in *G9a* mutants compared to wild-type flies. Upon infection with the DNA virus IIV-6, we detected only slight expression of these genes (1 to 4-fold, at 24 hpi and 7 dpi, when the replication plateau is reached), and expression levels were not significantly different between wild-type and *G9a* mutant flies (Fig 3H and 3I). We note that those viruses that induce higher Jak-Stat activation also induce higher mortality rates in *G9a* mutants (Fig 1B–1F). Our results are in line with a previous report showing that DCV, CrPV, DXV, and FHV, but not IIV-6, induce expression of the Jak-Stat dependent genes *vir-1* or *TotM* [7]. In that study, DXV induces strong *TotM* expression, and DCV, CrPV and FHV induce mainly *vir-1* expression, whereas under our experimental conditions, *TotA* and *TotM* are induced at higher levels than *vir-1* for all viruses.

Jak-Stat deficient flies were reported to display higher viral load and increased mortality upon DCV and CrPV infection [7,8], suggesting that the Jak-Stat pathway controls expression of antiviral effectors. Our data suggest that robust induction of Jak-Stat dependent genes is not sufficient for efficient host defense, which is in line with previous observations [8]. Moreover, the *G9a* phenotype seems counter-intuitive, since the antiviral Jak-Stat pathway is strongly activated in *G9a* mutant flies, yet they are hypersensitive to virus infection.

### *G9a* mutants display an altered transcriptional response to virus infection

**Fig 4:**
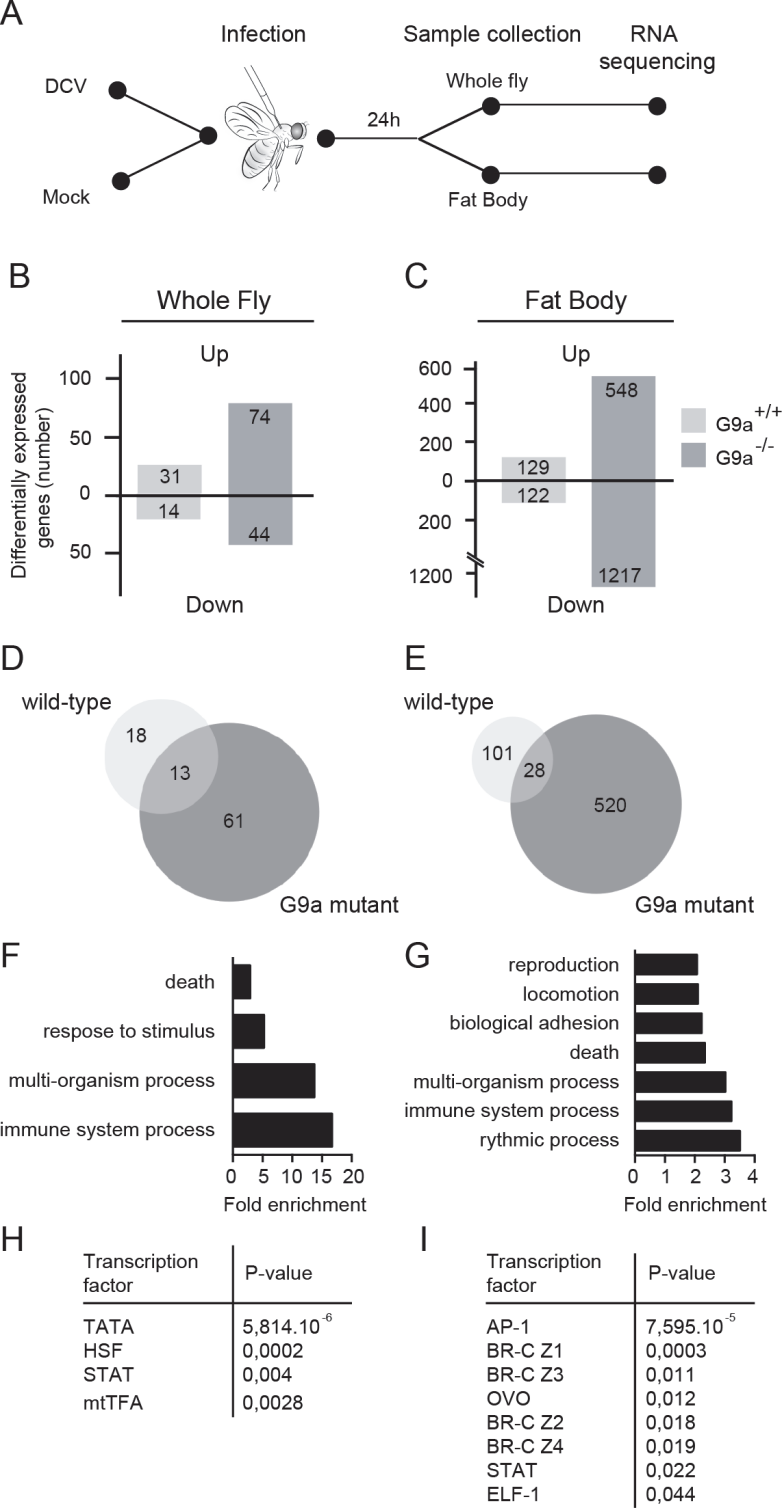
RNA sequencing and transcriptome analysis of wild-type and *G9a* mutant flies following DCV infection. (**A**) Experimental workflow. Three to five-day-old female flies were infected with DCV or mock-infected with Tris buffer as a control and total RNA was extracted for next-generation sequencing from whole flies or dissected fat bodies at 24 hpi. (**B,C**) Number of differentially expressed genes in (**B**) whole flies or (**C**) fat bodies of wild-type (*G9a* ^+/+^) or mutant (*G9a* ^-/-^) flies upon virus infection normalized to their respective mock control. Numbers of genes with ≥2-fold change are shown. (**D,E**) Venn diagrams representing the overlap of DCV-induced genes (relative to mock) between wild-type and *G9a* mutant flies in (**D**) whole flies or (**E**) fat bodies. (**F-I**) Gene ontology (GO) and predicted transcription factor binding sites of genes that are expressed at ≥2-fold higher levels in DCV infected *G9a* mutants than in infected wild-type flies. (**F**,**G**) All significantly enriched GO terms of level 2 are shown (*P* < 0.05 in a hypergeometric test with Benjamini & Hochberg correction). (**H,I**) Pscan was used to predict transcription factor binding sites in the 500-bp region upstream of the transcription start site using the TRANSFAC database. Significantly enriched transcription factors compared to the genome-wide mean are shown (*P* < 0.05 in a z-test). Data are from whole flies (**F,H**) or dissected fat bodies (**G,I**).

To analyze the transcriptional response to viral infection at a genome-wide scale, we performed transcriptome analyses by next-generation sequencing (RNA-seq). We infected wild-type or *G9a* mutant flies with DCV, and collected whole flies or dissected fat bodies at 24 hpi (Fig 4A). At this time point, flies do not yet exhibit pathological symptoms, such as reduced locomotion and abdominal swelling.

We first determined the number of differentially expressed genes (≥ 2-fold) upon DCV infection in whole fly (Fig 4B) or fat body (Fig 4C) relative to mock-infected flies. We noted that only a limited number of genes were induced upon DCV infection in whole wild-type flies (n = 31), whereas many more genes were induced in the fat body (n = 129), possibly because the fat body is a major immune organ and a target organ for DCV [25]. In *G9a* mutants, significantly more genes were induced upon DCV infection than in wild-type flies, both in whole flies and in dissected fat bodies (n = 74, *P* < 0.0001 and n = 548, *P* < 0.0001, respectively, Pearson’s chi-squared test). We also observed a large number of genes that were downregulated upon DCV infection. These genes followed the same trends as the virus-induced genes, with greater number of genes affected in *G9a* mutants both in whole fly and fat body. These observations are in agreement with the results from Fig 3 and suggest that the transcriptional response to virus infection is dysregulated in *G9a* mutants.

Only a limited number of genes were induced by DCV in both wild-type and *G9a* mutant flies (13 and 28 genes in whole fly and fat body, respectively; Fig 4D and 4E). This core set of virus-induced genes consisted of genes involved in stress responses such as heat shock proteins (*Hsp70 family*, *Hsp68*) and the Jak-Stat dependent Turandot proteins (*TotM*, *TotX*, *TotC*), as well as other Jak-Stat dependent genes, *Diedel* [32] and *Suppressor of Cytokine Signaling 36E* (*Socs36E*) [8], and genes of unknown function (S4A and S4B Fig).

### Jak-Stat dependent genes are enriched in the *G9a* transcriptome

We focused our subsequent analyses on the genes that were differentially expressed (≥2-fold) in *G9a* mutants, based on the prediction that if *G9a* represses genes by depositing H3K9me2 marks, direct target genes are most likely de-repressed in *G9a* mutants. To analyze whether specific biological processes are dysregulated in *G9a* mutants, we analyzed Gene Ontology (GO) terms of genes that were expressed at least 2-fold higher in DCV-infected *G9a* mutant flies over infected wild-type flies. In the whole fly dataset, we observed significant enrichment for GO terms, such as “response to abiotic stimulus" and "response to stress" (within the ancestral GO term "response to stimulus") and "immune response" (ancestral GO term "immune system process") (Fig 4F and S4C Fig). GO term analysis on the fat body dataset identified several additional processes, including "reproduction" and “locomotion” (Fig 4G and S4D Fig). Using Pscan [33] to predict transcription factor binding sites in the promoter regions of the differentially expressed genes, we observed, in addition to the TATA-box binding motif, strong enrichment of Stat binding sites, and target sites of the JNK cascade transcription factor, AP-1 (Fig 4H and 4I). In accordance, we noted among the categories “response to stress” and “immune system processes” genes of the Jak-Stat and c-Jun N-terminal Kinase (JNK) signaling pathways, which included pathways components (*dPIAS*, *Socs36E* for Jak-Stat; *Hemipterous*, *Gadd45*, *Jra*, *Kay* for JNK) as well as some of their downstream targets (*Socs36E*, *vir-1*, *CG13559*, *CG1572* for Jak-Stat; *Puckered* and *Rab-30* for JNK) [34].

### *G9a* targets genes of the Jak-Stat pathway

Our results indicate that the transcriptional response to infection is highly dysregulated in the absence of *G9a* and that Jak-Stat pathway components and downstream targets are among the genes that are derepressed in *G9a* mutants. We then asked whether these derepressed Jak-Stat genes are direct targets of *G9a*, or whether they are affected indirectly.

**Fig 5:**
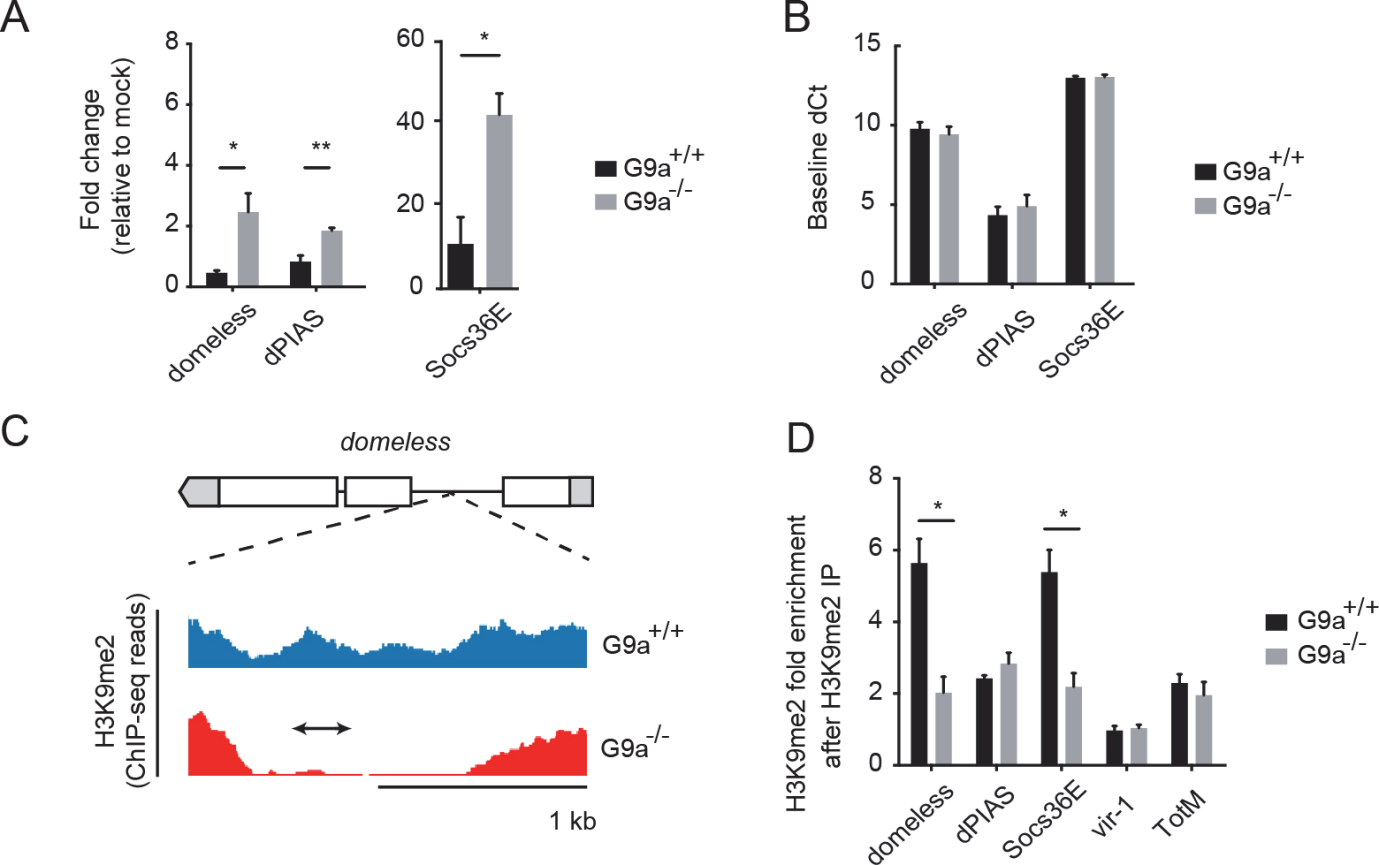
*G9a* targets genes of the Jak-Stat pathway. (**A**) Expression levels of *domeless*, *dPIAS*, and *Socs36E* at 24 hpi in fat bodies of 3 to 5-day-old female wild-type or *G9a* mutant flies challenged with DCV (10,000 TCID_50_ units). Data are expressed as fold change relative to mock infection (Tris buffer). (**B**) Basal expression levels of Jak-Stat genes measured by RT-qPCR on fat bodies of 3 to 5-day-old unchallenged female wild-type and *G9a* mutant flies. Basal expression is presented as dCt (difference between Ct of the gene of interest and the Ct of *Ribosomal Protein 49)*. (**C**) Representative example of a *G9a* target locus within the *domeless* gene, defined as a genomic region in which the H3K9me2 mark is present in wild-type flies, but not in *G9a* mutants, in a previous study [20]. Blue and red plots represent sequence reads in H3K9me2 ChIP-seq analyses of wild-type and *G9a* mutants, respectively [20]. Gene structure is indicated with boxes for exons, lines for introns, and gray boxes for untranslated regions. The arrow represents the position of the amplicon generated by qPCR after Chromatin-Immunoprecipitation (ChIP-qPCR). (**D**) H3K9me2 ChIP-qPCR on fat bodies of wild-type or *G9a* mutant flies. Fold enrichment is the percentage of input of the gene of interest normalized to that of a reference gene with very low H3K9me2 marks (*moca*). Specificity control experiments for ChIP-qPCR experiments are shown in S5E–S5J Fig. Data are means and s.d. of (**A,B**) three independent pools of at least 10 fat bodies, or (**D**) three independent pools of 80 female fat bodies, for each genotype. Data are from one experiment representative of 2 (**A**,**B**) or 6 (**D**) independent experiments. *P < 0.05 (Student’s t-test).

A previous study identified putative *G9a* target sites by comparing genome-wide H3K9me2 profiles obtained by chromatin immunoprecipation (ChIP) followed by next generation sequencing in wild-type and *G9a* mutant larvae [20]. Interestingly, these predicted targets are enriched for the GO term "Jak-Stat cascade" (*P* = 0.0011, 2.3-fold enrichment). We therefore selected Jak-Stat genes that fulfilled three criteria for further analysis: i) harboring a reported loss-of-methylation site in *G9a* mutants, ii) previously shown to be involved in defense responses, iii) being upregulated in the transcriptome sets of challenged *G9a* mutants. This set of five genes consisted of pathway components and regulators (*domeless*, *dPIAS*, *Socs36E*), as well as the downstream targets *vir-1* and *TotM* [10,35–38]. Using RT-qPCR, we confirmed that all five predicted *G9a* target genes show over-induction in response to virus infection in *G9a* mutant fat bodies (*domeless*, *dPIAS*, *Socs36E*, Fig 5A *; vir-1*, *TotM*, Fig 3B). For none of these genes, a difference in basal expression was observed in the absence of viral infection, indicating that these genes are only derepressed upon viral infection in *G9a* mutants (Fig 5B and Fig 3D).

We next analyzed *G9a*-dependent targeting of these Jak-Stat genes by H3K9me2 ChIP followed by qPCR (ChIP-qPCR) in dissected fat bodies of wild-type and *G9a* mutants. We designed qPCR primers in the loss-of-methylation regions observed in ChIP-seq, as shown (*domeless* in Fig 5C; *dPIAS*, *Socs36E*, *vir-1*, *TotM* in S5A–S5J Fig). We found that *Socs36E* and *domeless* were significantly depleted of H3K9me2 in the fat body of *G9a* mutants at previously predicted *G9a* target sites [20] (Fig 5D). Not all *G9a* targets sites could be confirmed, possibly because ChIP-seq and ChIP-qPCR have been performed at different developmental stages and tissues (whole larvae versus adult fat body, respectively). Although we could not confirm direct targeting by *G9a* of *dPIAS*, *vir-1* and *TotM* using ChIP-PCR, we did observe higher expression of these genes in infected *G9a* mutants. Upregulation of these genes could be a secondary effect resulting from the dysregulation of pathway components, such as *domeless* and *Socs36E*, in *G9a* mutants, rather than from direct epigenetic regulation by *G9a*. Taken together, these observations suggest that *G9a* epigenetically regulates a subset of Jak-Stat genes in the adult fat body to shape their transcriptional response to virus infection.

### *G9a* regulates tolerance through modulation of Jak-Stat pathway activity

Our data suggest that *G9a* regulates Jak-Stat responses to prevent excessive expression of downstream target genes. We performed genetic epistasis tests to analyze the relationship between *G9a* and the Jak-Stat pathway. Epistasis is defined as a genetic interaction in which a mutation in one gene masks the phenotype of a mutation in another gene. We hypothesized that if *G9a* mediates viral tolerance through dampening Jak-Stat-induced transcription, inactivation of the Jak-Stat pathway would mask the hypersensitivity of *G9a* mutants to virus infection. Alternatively, if *G9a* confers tolerance to DCV infection in a Jak-Stat independent manner, simultaneous loss of *G9a* and Jak-Stat function would result in more dramatic hypersensitivity to virus infection.

**Fig 6:**
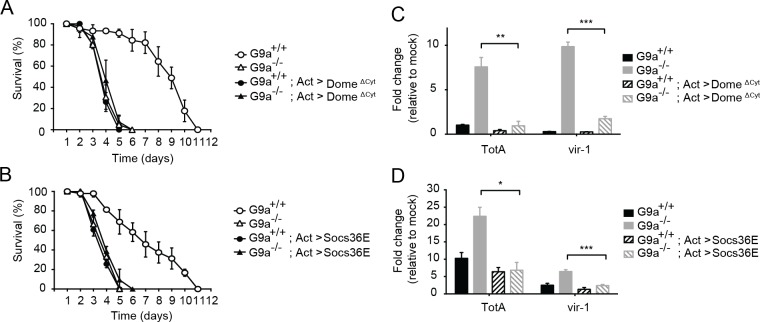
Genetic interaction between *G9a* and the Jak-Stat pathway. (**A,B**) Survival upon DCV infection (1,000 TCID_50_ units) of wild-type or *G9a* mutant and wild-type flies overexpressing (**A**) a dominant negative version of the *domeless* receptor (dome^ΔCyt^), or (**B**) the negative regulator of Jak-Stat signaling *Socs36E*. The UAS/Gal4 system was used to drive transgene expression. Gal4 is expressed under control of the actin promoter (*Act-Gal4*) to drive ubiquitous expression of the *UAS-dome* ^ΔCyt^ and *UAS-Socs36E* transgenes. Control flies expressing only the *Act-Gal4*, the *UAS-dome* ^ΔCyt^, or the *UAS-Socs36E* transgenes were included as controls (see S5A and S5B Dataset). Mock infections where performed along the experiments and are shown in S6A and S6B Fig. (**C,D**) Expression of *TotA* and *vir-1* upon DCV infection of wild-type or *G9a* mutant flies, expressing (**C**) dome^ΔCyt^, or (**D**) *Socs36E*. Expression of the gene of interest (by RT-qPCR) was normalized to transcript levels of the housekeeping gene *Ribosomal Protein 49* and expressed as fold change relative to mock infection (Tris buffer). Data are means and s.d. of three independent pools of at least 15 male flies for each genotype. (**A**,**B**) A representative experiment of two independent experiments is shown. Differences in expression of *TotA* and *vir-1* were evaluated with a Student’s t-test (*P < 0.05; ** *P* < 0.01; *** *P* < 0.001).

To test our hypothesis, we combined the mutant *G9a* allele with a dominant negative allele of the Jak-Stat pathway receptor *domeless* (*dome* ^ΔCyt^) under control of a UAS enhancer [39]. We drove expression of *dome* ^ΔCyt^ in the background of *G9a* mutants and wild-type controls using the ubiquitous actin-Gal4 driver and challenged flies with DCV. As expected [8], overexpression of *dome* ^ΔCyt^ increased mortality rates in a *G9a* ^+/+^ background. Remarkably, the difference in survival between *G9a* ^-/-^ and *G9a* ^+/+^ flies was masked in the Jak-Stat impaired genetic background (Fig 6A). Moreover, mortality rates of double mutant flies (G9a^-/-^ and Jak-Stat deficient) were similar to those of flies in which either *G9a* or Jak-Stat was inactivated. Therefore, our data suggests a genetic interaction between *G9a* and the Jak-Stat pathway receptor, *domeless*. Additionally, we found that *dome* ^ΔCyt^ negated the over-induction of *TotA* and *vir-1* in DCV-infected *G9a* mutants, demonstrating that *G9a* regulates these genes in a Jak-Stat dependent manner (Fig 6C).

To confirm these results with another Jak-Stat loss-of-function allele, we performed a second epistasis experiment using a fly strain overexpressing the negative regulator of the Jak-Stat pathway *Socs36E* under control of the UAS sequence [40]. Similar to the experiment with *dome* ^ΔCyt^, overexpression of *Socs36E* masked the hypersensitivity phenotype of *G9a* mutants to virus infection, suggesting a genetic interaction between *G9a* and *Socs36E* (Fig 6B). Again, as expected, *Socs36E* overexpression significantly reduced expression of *TotA* and *vir-1* (Fig 6D). In both assays, mock infections were performed in parallel, confirming that differences in survival cannot be attributed to the injury caused by the injection itself (S6A and S6B Fig). As the DCV inoculum of 1,000 TCID_50_ induced high mortality rates in *G9a* mutants, as well as in Jak-Stat deficient flies, it remained possible that we may have missed higher mortality rates in flies carrying both mutations. Therefore, we repeated the epistasis experiments using a lower inoculum of 100 TCID_50_, and confirmed that combining Jak-Stat inactivation with *G9a* loss-of-function did not yield higher mortality rates than in single mutants (S6C and S6D Fig).

In both cases, inhibition of Jak-Stat signaling in wild-type flies masked the effect of a *G9a* null mutation upon viral challenge, indicating a genetic interaction between *G9a* and the Jak-Stat components. Taken together, these results suggest that *G9a* regulates viral tolerance through modulation of Jak-Stat pathway activity.

### Jak-Stat hyperactivation induces early mortality after virus infection

Our results suggest that *G9a* buffers Jak-Stat dependent responses to prevent excessive expression of Jak-Stat dependent genes. We hypothesize that hyperactivation of the Jak-Stat response induces immunopathology that causes increased mortality of *G9a* mutants upon virus infection. This hypothesis predicts that ectopic activation of the Jak-Stat pathway results in increased rates of mortality upon virus infection.

**Fig 7:**
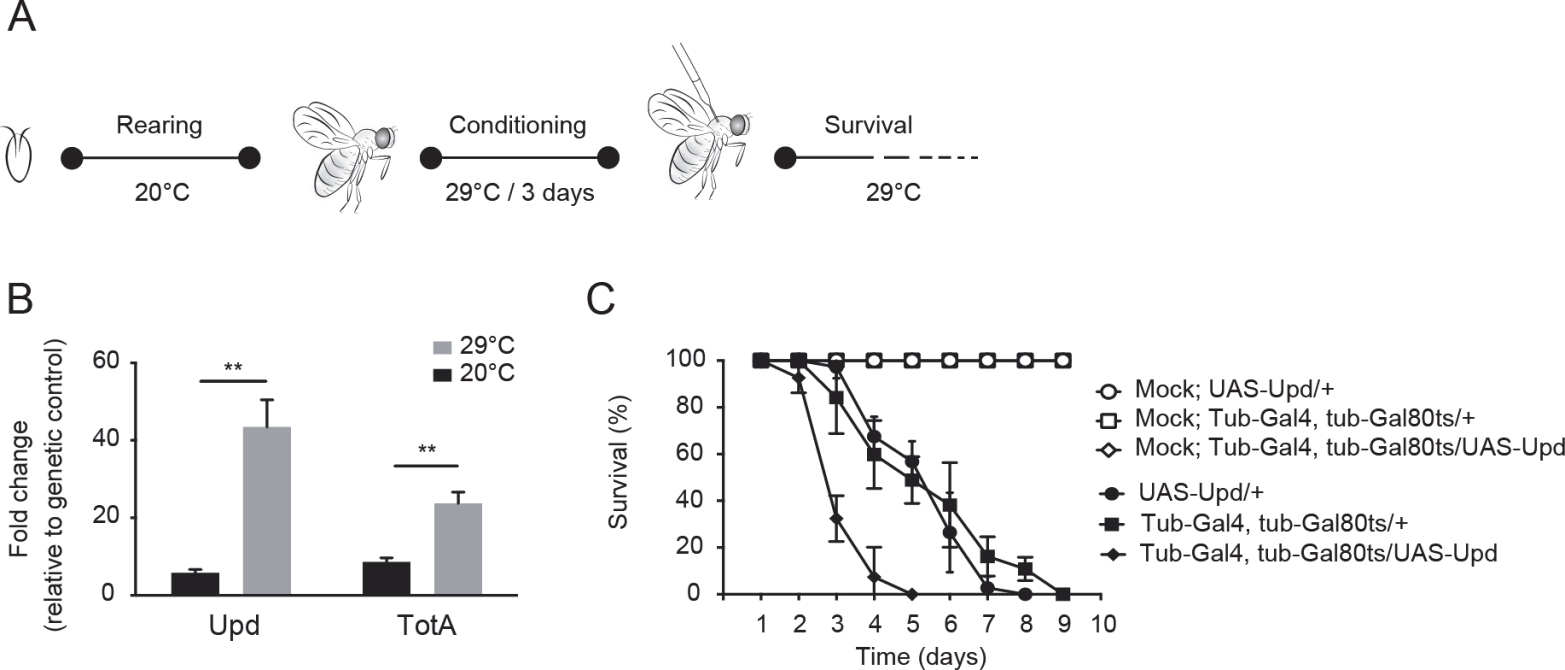
Hyperactivation of the Jak-Stat pathway renders flies hypersensitive to virus infection. (**A**) Experimental set-up. Expression of the *Upd* transgene was induced specifically in adult flies using the *Gal4/Gal80ts* system. *Gal80ts* is a temperature-sensitive allele of the Gal80 inhibitor that binds Gal4 to prevent activation of gene expression at 20°C. At 29°C, Gal80ts is degraded, allowing Gal4 to bind to the Upstream Activating Sequences (UAS) to induce gene expression. Flies were reared at 20°C, and 0 to 3-day-old adults were conditioned at 29°C for 3 days prior to viral challenge. (**B**) Expression levels by RT-qPCR of *Upd* and *TotA* in flies carrying the temperature-dependent *Upd* overexpression system (*UAS-Upd; tubulin-Gal4/Gal80ts*) after 3 days conditioning at 29°C. The *Gal4* and *Gal80ts* transgenes were combined with the *UAS-Upd* by standard genetic crosses at 20°C and 0 to 3-day-old adult offspring was cultured for 3 days at 20°C or at 29°C before RNA levels were analyzed by RT-qPCR. Transcript levels of *Upd* and *TotA* were normalized to RNA levels of the housekeeping gene *Ribosomal Protein 49*, and expressed as fold change relative to control flies carrying only the *UAS-Upd* transgene. (**C**) Survival of flies carrying the temperature-dependent *Upd* overexpression system (*UAS-Upd; tubulin-Gal4/Gal80ts*) and genetic control flies upon DCV infection (1,000 TCID_50_ = units) at 29°C. Data are means and s.d. of three independent pools of at least 10 male flies for each genotype. Data in (**C**) are from one experiment representative of 2 independent experiments. Differences in expression of *Upd* and *TotA* were evaluated with a Student’s t-test (*P < 0.05; ** *P* < 0.01; *** *P* < 0.001).

To test this prediction, we activated the Jak-Stat pathway in adult flies by ubiquitous expression of *Unpaired* (*Upd*), a ligand for the *domeless* receptor, and subsequently infected flies with DCV. Since the Jak-Stat pathway has important functions in development, we used the temperature sensitive *Gal80ts* allele [41] to induce ubiquitous *Upd* expression in adult flies by transferring them from 18–20°C (non-permissive temperature) to 29°C (permissive temperature) (Fig 7A). We confirmed by RT-qPCR that *Upd* as well as the Jak-Stat target gene *TotA* were strongly induced at 3 days after the shift to 29°C (Fig 7B). We next challenged *Upd*-overexpressing adult flies with virus. Strikingly, flies with a hyperactivated Jak-Stat pathway succumbed earlier to DCV infection (mean survival = 3.3 days) than genetic control flies expressing only the *UAS-Upd* transgene or the *tubulin-Gal4*, *tubulin-Gal80ts* drivers (mean survival = 5.5 days for both, *P* < 0.001) (Fig 7C). Moreover, irrespective of the genotype, mock infection did not induce mortality, excluding the possibility that incubation at 29°C is a stressor that triggers early lethality. We conclude that ectopic Jak-Stat activation phenocopies loss of *G9a*, indicating that immune hyperactivation may underlie the hypersensitivity of *G9a* mutant flies to DCV infection.

## Discussion

Disease tolerance was recently defined as a defense strategy that reduces the negative impact of infection on host fitness, without a concomitant reduction of pathogen burden [3,4]. The concept of tolerance (also termed resilience) provides an exciting, novel perspective on pathogen-host interactions in metazoans. A few examples of tolerance to bacterial or viral infections have been described in flies [42–48], but the mechanisms of tolerance remain largely unknown. In this study, we elucidate a novel epigenetics-based mechanism for tolerance. We provide evidence that the histone methyltransferase *G9a* contributes to tolerance by regulating the antiviral Jak-Stat signaling pathway.

*G9a* mutant flies are hypersensitive to RNA virus infection. Transcriptome analyses indicate that Jak-Stat pathway genes are highly upregulated upon DCV challenge in *G9a* mutants, whereas their basal levels prior to viral infection are normal. This phenotype, like others reported previously [49,50], seems paradoxical: the antiviral Jak-Stat pathway is strongly activated, yet *G9a* flies are hypersensitive to infection, showing that immune induction *per se* is not sufficient for efficient host defense. We propose that increased expression of Jak-Stat dependent genes causes immunopathology, eventually resulting in earlier mortality upon virus infection. In support of this hypothesis, we demonstrated that *G9a* limits the strength of the immune response through Jak-Stat and that ectopic hyperactivation of Jak-Stat signaling triggered early lethality after DCV infection, thus phenocopying the *G9a* phenotype. Therefore, we propose that epigenetic regulation by *G9a* dampens Jak-Stat signaling to avoid immune hyperactivation and subsequent mortality.

*G9a* seems to be required for tolerance to RNA viruses, but not to DNA viruses. *G9a* mutants induce higher expression of the Jak-Stat dependent genes *vir-1*, *TotA*, and *TotM* and show increased lethality rates upon infection with four RNA viruses (DCV, CrPV, FHV, and DXV). A previous study found that these viruses all induce either *vir-1* or *TotM* to some extent, but that a resistance phenotype for Jak-Stat mutants (higher lethality rates in combination with increased viral load) was only observed after DCV and CrPV infection [7]. Thus, whereas Jak-Stat is only required for resistance to DCV and CrPV infection, our results suggest that all RNA viruses activate the Jak-Stat pathway and that precise epigenetic control of the pathway is required to prevent immunopathology.

Like in mammals, hyperactivation of immune pathways in *Drosophila* is detrimental for fitness and survival. For instance, overexpression of antimicrobial peptides or loss of negative regulators such as *Caudal* or the catalytic peptidoglycan receptor proteins (*PGRP-LB* and *PGRP-SCs*) triggers severe tissue pathology in the gut that are reminiscent of chronic inflammatory syndromes in mammals [49,51]. The mechanism by which Jak-Stat overactivation triggers lethality remains to be determined, but may involve expression of potentially toxic gene products that require tight regulation. Moreover, we cannot exclude that additional derepressed genes upon loss of *G9a* contribute to increased mortality of mutant flies. Alternatively, the *G9a* phenotype might be caused by defects in cell growth, differentiation, tissue homeostasis or apoptosis, which are also under control of the Jak-Stat pathway. We do note, however, that an external infectious stimulus, i.e. virus infection, was required to cause increased mortality upon genetic hyperactivation of the Jak-Stat pathway, and that *G9a* mutants appear to develop normally, thus excluding more generalized defects.

Our transcriptome analysis uncovered that, in addition to the Jak-Stat pathway, a multitude of pathways are activated by virus infection, many of which are of interest for follow-up studies. We observed a strong activation of the JNK pathway upon DCV infection. In accordance, predicted binding sites for the AP-1 complex, the transcriptional module of the JNK pathway, were highly enriched in promoters of genes upregulated upon DCV infection in *G9a* mutant fat bodies. Whether Stat and AP-1 associate upon virus infection to regulate immune genes cooperatively, as previously described in lipopolysaccharide stimulated *Drosophila* cells [52,53], is an interesting question for future investigation.

Our study makes an important contribution to understanding tolerance mechanisms beyond *Drosophila*. Two *EHMT/G9a* paralogs exist in mammals, *EHMT1/GLP* and *EHMT2/G9a* [20]. They form a heterodimeric complex, and loss of either protein results in nearly identical phenotypes [54]. We analyzed published microarray data of mice in which the *G9a* and *GLP* genes were inactivated in forebrain neurons and observed enrichment for the GO term "immune response", and over-representation of NF-κB binding sites in differentially regulated genes, suggesting that *G9a* also regulates immune signaling cascades in mammals (S7 Fig). Indeed, a previous study suggested that the *G9a*-dependent H3K9me2 mark is an epigenetic determinant of the interferon response in murine and human cells [55]. In that study, the abundance of H3K9me2 at the promoters of the Interferon-β (*Ifnβ*) gene and Interferon stimulated genes (ISG) correlates with expression levels of these genes in different cell types, but deficiency in *G9a* did not affect basal gene expression. Pharmacological inhibition or genetic ablation of *G9a* increased *Ifnβ* and ISG expression in mouse fibroblasts and rendered these cells resistant to viral infection.

Our results demonstrate that the role of *G9a* in controlling the responsiveness to immune challenge is evolutionarily conserved. Moreover, while the *in vitro* cell culture model suggested that loss of *G9a* would be beneficial to the antiviral response of the host [55], our data show that loss of *G9a* disrupts tolerance mechanisms at the organismal level, and is therefore detrimental for survival. This seems to better match the observations in humans. Heterozygous loss of *EHMT1/GLP* causes Kleefstra syndrome (OMIM number 610253). This rare disorder is characterized by developmental delay and severe intellectual disability. Interestingly, up to 60% of Kleefstra syndrome patients suffer from recurrent infections; yet, these patients do not suffer from primary immune deficiencies [56]. Whether defects in tolerance explain this aspect of the clinical presentation of Kleefstra syndrome remains an interesting hypothesis.

## Materials and Methods

### Fly strains and husbandry

Flies were reared on standard cornmeal-agar media at 25°C on a light/dark cycle of 12h/12h. *G9a* ^DD2^ mutants were generated previously by mobilization of the P-element *KG01242* located in the 5’ UTR of the gene [20]. *G9a* ^DD2^ has been used throughout the main text and is referred to as *G9a* ^-/-^. A precise transposon excision line, referred to as *G9a* ^+/+^, has been generated in the same genetic background and serves as a control in all experiments. An independent null allele, *G9a* ^DD3^, has been generated by mobilization of the same P element and contains a deletion of 1850 bp that spans the translation start site [20] (S1B Fig). The following fly stocks and alleles have been described before: *UAS-G9a* (ref. [20]), *C564-Gal4* fat body driver (ref. [57]), *Hml-Gal4* hemocyte driver (ref. [58]), *UAS-dome* ^ΔCYT^ (ref. [8,37]), *UAS-Socs36E* (ref. [59]), *UAS-Upd* (ref. [59]), *tubulin-Gal4*, *tubulin-Gal80ts* (ref. [60]), and *Argonaute 2* ^414^ (ref. [61]). The driver lines *armadillo-Gal4* and *repo-Gal4* were obtained from the Bloomington Stock Center. *In vivo* RNAi experiments were performed by crossing GMR-Gal4, UAS-*th* ^RNAi^/CyO male flies [26] with *G9a* ^+/+^or *G9a* ^-/-^ virgins. The eye phenotype was monitored in two to four-day-old male F1 offspring lacking the CyO balancer. *Upd* was conditionally overexpressed by crossing *tubulin-Gal4*, *tubulin-Gal80ts* with *UAS-Upd* flies. Flies were reared at 20°C until hatching. Zero to three-day-old F1 offspring were then incubated at 29°C for 3 days prior to viral challenge, and cultured at 29°C throughout the remainder of the experiment.

### Virus infection

Fly stocks were raised for two generations on standard fly flood containing 0.05 mg/ml tetracycline hypochloride (Sigma) to clear *Wolbachia* infection. Absence of *Wolbachia* was verified by PCR on DNA of whole flies using *Wolbachia*-specific primers, as described previously [24]. Persistent virus infections were cleared by bleaching embryos, and absence of DCV, DAV and Nora virus was verified by RT-PCR, as previously described [24].

Virus stocks were prepared as described [24]. Three to five-day-old flies were anesthetized with CO_2_ and injected with virus suspension using a Nanoject II injector (Drummond) in the thorax, between the mesopleura and the pteropleura. Virus suspensions in 10 mM Tris-HCl, pH 7.3 contained 1,000 median tissue culture infectious dose (TCID_50_) of DCV and CrPV; 14,000 TCID_50_ of IIV-6; 3,000 TCID_50_ of FHV and 2,000 TCID_50_ of DXV for all survival experiments. 10,000 TCID_50_ of DCV was used in experiments in which transcriptional responses were analyzed. Flies were cultured at 25°C and transferred to fresh food every 3 days. Survival was monitored daily; lethality at day 1 was attributed to the injection procedure and subtracted from the survival analysis. Unless noted otherwise, three pools of 10 to 15 flies were injected per condition with independent dilutions of virus stock. Fat body tissues were isolated by careful dissection of the abdominal carcasses of adult flies and removal of the gut and reproductive system. This procedure recovers cuticle-associated fat body with minor contamination by muscular and epidermal tissues [62].

### Virus titration

Drosophila S2 cells (Invitrogen) were cultured at 25°C in Schneider’s *Drosophila* Media (Gibco) supplemented with 10% heat-inactivated Fetal Calf Serum (PAA), 50 U/mL Penicillin and 50 μg/mL Streptomycin (Gibco). DCV titers were determined by end-point dilution, as described previously [24]. Briefly, 2.10^4^ cells were seeded in 96-well plates and ten-fold dilutions of fly homogenate were inoculated in quadruplicate. Cells were transferred to fresh medium at day 5, and cytopathic effect (CPE) was monitored until day 14. Viral titers were calculated according to the method of Reed and Muench [63].

### RNA analysis

RNA was isolated from flies using Isol-RNA lysis Agent (5-Prime), treated with DNase I (Ambion), and cDNA synthesis was performed on 1 μg RNA using TaqMan Reverse Transcription Reagents (Applied Biosystems) according to the manufacturer’s instructions. qPCR was performed on a LightCycler 480 using SYBR Green I Master Mix (Roche). The qPCR program was the following: 95°C for 5 min, and 45 cycles of 95°C for 5s, 60°C for 10s, 72°C for 20s. Expression of the gene of interest was normalized to transcript levels of the housekeeping gene *Ribosomal Protein 49* (Rp49). The following primers were used for qPCR:

Rp49 forward, 5’- ATGACCATCCGCCCAGCATAC-3’;

Rp49 reverse, 5’-CTGCATGAGCAGGACCTCCA-3’;

Vago forward, 5’- CAGCCAAGCGATTCCTTATC-3’;

Vago reverse, 5’- CTCATACAGTGGGCAGCATC-3’;

vir-1 forward, 5’-ATTACTCCGAATTCGAAGCTTCC-3’;

vir-1 reverse, 5’- CGAATTCTTCACGCTCCTTC-3’;

Listericin forward, 5’-TTGCGGCCATTCTGGCCATG-3’,

Listericin reverse, 5’- TTTACGTCCCCAACTGGAAC-3’;

TotA forward, 5’- CCCTGAGGAACGGGAGAGTA-3’;

TotA reverse, 5’- CTTTCCAACGATCCTCGCCT-3’;

TotM forward, 5’- ACCGGAACATCGACAGCC-3’;

TotM reverse, 5’- CCAGAATCCGCCTTGTGC-3’;

Drosomycin forward 5’-GTACTTGTTCGCCCTCTTCG-3’;

Drosomycin reverse, 5’- ACAGGTCTCGTTGTCCCAGA-3’;

Metchnikowin forward 5’- TACATCAGTGCTGGCAGAGC-3’;

Metchnikowin reverse, 5’- AATAAATTGGACCCGGTCTTG-3’;

Diptericin forward, 5’- TGTGAATCTGCAGCCTGAAC-3’;

Diptericin reverse, 5’- GCTCAGATCGAATCCTTGCT-3’;

DCV forward, 5’- TTGCCATTGCACCACTAAAA -3’;

DCV reverse, 5’- AAAATTTCGTTTTAGCCCAGAA -3’;

Domeless forward, 5’- AGCTCTGATCCGGATTGTTG-3’;

Domeless reverse, 5’-ATCTCACCGCATTCACCAAG-3’;

dPIAS forward, 5’-AACTGCCCTGTATGCGACAA-3’;

dPIAS reverse, 5’-ACACCTCCTGGAAGTAGCCA-3’;

Socs36E forward, 5’-GTTGCTGCTCCCATTGAAAG-3’;

Socs36E reverse, 5’-GCAAAAGTCGGAGTGTGAGAG-3’;

### *In vivo* RNAi reporter assay

RNAi competency of adult flies was analyzed using a reporter assay that was adapted from a previously published method in S2 cells [27,28]. In vivo plasmid transfection was based on a method described for *Aedes aegypti* mosquitoes [64,65]. Three to five-day-old female flies were injected in the abdomen with a 100 nl suspension containing a 1:1 mixture of Schneider’s *Drosophila* Media (Gibco) and Lipofectamine 2000 (Invitrogen) complexed with 80 ng pMT-GL3 (encoding firefly luciferase, FLuc), 50 ng pMT-Ren (encoding *Renilla* luciferase, RLuc) and 1 ng FLuc-specific or non-specific control dsRNA. After incubation for 3 days at 25°C, flies were homogenized with a Douncer in passive lysis buffer (Promega). Supernatant was collected after 10 min centrifugation at 16,000 × g and transferred to a new tube, followed by centrifugation for 5 min at 16,000 × g. 25μL of fly lysate was used to measure FLuc and RLuc activity using the Dual Luciferase assay reporter system (Promega). Ratios of FLuc/Rluc were calculated for each sample, and data are presented as fold silencing relative to the non-specific dsRNA control (GFP).

### Chromatin immunoprecipitation followed by qPCR

Eighty dissected fat bodies were homogenized in PBS with a douncer and crosslinked with 3.7% formaldehyde for 30 minutes at room temperature. The cross-linking reaction was quenched by addition of 1.25 mM glycine, and the samples were washed with 1 mL PBS and resuspended in a buffer containing 150 mM Tris-HCl (pH 7.5), 600 mM KCl, 150 mM NaCl, 10 mM EDTA, 1 mM EGTA, 1.5 mM spermine (Sigma) and 5 mM spermidine (Sigma). Tissue was further homogenized using a QiaShredder column, and cells were lysed by adding the same buffer supplemented with 2% Triton-X100. Nuclei were pelleted by centrifugation at 6,000 rpm for 10 min, and resuspended in 250 μL incubation buffer (0.75% SDS, 5% Triton-X100, 750 mM NaCl, 5mM EDTA, 2.5 mM EGTA, 50 mM Tris pH 8.0, 0.4% BSA, 1x protease inhibitor cocktail complete (Roche)). After nuclei purification, chromatin was sonicated at 4°C using a Bioruptor sonicator (Diagenode) for 30 minutes at high power with cycles of 30 seconds ON, and 30 s OFF. Anti-H3K9me2 (ab1220, Abcam), anti- H3 (ab1791, Abcam), anti-V5 (R960-20, Invitrogen) antibodies, and Prot A/G beads (Santa Cruz) were used to capture antibody-bound chromatin overnight at 4°C on a rotating wheel. Chromatin was eluted and de-crosslinked for 4 hours at 65°C in 416 μL elution buffer containing 0.2 M NaCl, 1% SDS and 0.1 M NaHCO_3_. DNA was then isolated using phenol/chloroform, precipitated overnight at -20°C with 1 mL 100% ethanol, 5 μg linear acrylamide, 0.1 M NaAc, pH 5.2. The pellet was washed with 70% ethanol and resuspended in water. Non-immunoprecipitated DNA was isolated in parallel from purified nuclei and used as an input control in qPCR.

qPCR was performed on a LightCycler 480 using SYBR Green I Master Mix (Roche) using the following qPCR program: 95°C for 10 min, and 40 cycles of 95°C for 15s, 60°C for 1 min. The percentage of immunoprecipitated DNA relative to the input was calculated after qPCR. Fold enrichment in H3K9me2 positive DNA was calculated by normalizing the percentage of input of the gene of interest to the euchromatic control gene previously shown to lack H3K9me2, *moca* [66]. We confirmed in our conditions that H3K9me2 marks are indeed nearly absent on *moca* in both wild-type flies and *G9a* mutants. Also, we show that histone H3 levels are identical between *G9a* mutant and wild-type flies, both on *moca* and *domeless*. Using an aspecific IgG isotypic control antibody, we verified very low aspecific background binding to chromatin (S5E–S5J Fig).

Primers for qPCR were designed in regions previously shown to be depleted of H3K9me2 in *G9a* mutants by ChIP-sequencing [20]. Sequences are as follows:

Socs36E forward, 5’-GAAATCCGATGTGCTGAAG-3’;

Socs36E reverse, 5’-ACATGGGGGTGTTTTACAGG-3’;

Domeless forward: 5’-CACGTGGATCCAAAATACCC-3’;

Domeless reverse, 5’-GATTGCGATTCCGAGAACTG-3’;

dPIAS forward, 5’- CACTGACTCAACCACGCTTC-3’;

dPIAS reverse, 5’-CCGTAAAAGGTGAACCGAAA-3’;

vir-1 forward, 5’- TTGTTCTGGGGCAGAGAAAG-3’;

vir-1 reverse, 5’- ATCGCTTCATGTCAGTGTCC-3’;

TotM forward, 5’-TTCGGGACGGTCACAGATAG-3’;

TotM reverse, 5’-TCTCGAAAAACCCCTGTAGC-3’;

### RNA sequencing

Thirty whole flies or 100 fat bodies of three to five-day-old flies were collected at 24 hours after infection with 10,000 TCID_50_ of DCV. Samples were frozen on dry ice and stored at -80°C before RNA was isolated using Isol-RNA Lysis reagent as described above. The cDNA library was prepared with the Illumina TruSeq mRNA kit and single-end sequencing was performed on an Illumina HiSeq 2000 (Baseclear BV, Leiden, the Netherlands). RNAseq was performed on a single biological replicate, and should be considered an exploratory analysis.

The FastQ sequence reads were generated in the Illumina Casava pipeline version 1.8.0. Initial quality assessment was based on data passing the Illumina Chastity filter. Reads containing only adapters or PhiX control sequences were removed by filtering protocols developed by Baseclear BV. The second quality control on the remaining reads was performed with FastQC quality control tool 0.10.0. Reads were mapped to the reference genome (*Drosophila melanogaster* R5/dm3, released in April 2006, UCSC Bioinformatics) using TopHat version 1.4.0. Differential expression between two datasets was analyzed with the Genomatix analysis suite (using DESeq 1.0.6). Gene Ontology enrichment was analyzed using GoToolBox [67], with a hypergeometric test with Benjamini & Hochberg correction. Level 2 GO terms are shown in Fig 4, and level 3 GO terms in S4 Fig Fold enrichment is the ratio of the GO term frequency in the *G9a* datasets to the genome-wide GO term frequency. Promoter binding-sites for transcription factors were predicted with Pscan [33] on the 500-bp region upstream of the transcriptional start site using the TRANSFAC database. Venn diagrams were generated using Biovenn [68]. The RNA-Seq data are available at the NCBI Gene Expression Omnibus under series accession number GSE56013.

### Statistical analysis

Kaplan-Meier analyses and log-rank tests, as implemented in SPSS Statistics (version 20, IBM), were used to evaluate whether differences in survival were statistically significant. For all other experiments, unpaired two-tailed Student’s t-tests and Pearson’s chi-squared test, as implemented in Graphpad Prism version 6, were used to determine statistical significance. *P*-values below 0.05 were considered statistically significant.

## Supporting Information

S1 Fig Hypersensitivity of *G9a* mutants to RNA virus infection is not sex-dependent, or allele-specific. (**A**) Survival of male, wild-type or *G9a* mutant flies upon DCV infection, or Tris buffer control (mock). The mean survival is 8.9 days for wild-type flies, and 5.1 days for *G9a* mutants (*P* < 0.001). (**B**) Structure of the *G9a* locus. Boxes represent exons (5’ and 3’-untranslated regions in gray, and coding sequence in white). The KG01242 P-element insertion site that was used to generate the *G9a* ^DD3^ allele is depicted by dashed lines. Size and location of the *G9a* deletion in the *G9a* ^DD3^ allele are indicated. (**C,D**) Survival of wild-type or *G9a* ^DD3^ mutants infected with (**C**) DCV, (**D**) IIV-6, or with Tris buffer as a control (mock). Upon DCV infection (**C**), the mean survival is 6.9 days for wild-type flies, and 4.4 days for *G9a* ^DD3^ mutants (*P* < 0.001). Data represent means and s.d. of three biological replicates of 15 male flies (**A**) or 20 female flies (**C**,**D**) per replicate for each genotype. A representative experiment of 3 independent experiments is shown.
(TIF)

S2 Fig Expression of a *G9a* transgene in hemocytes or glia does not rescue virus hypersensitivity of *G9a* mutants. (**A,B**) Survival of wild-type or *G9a* ^-/-^ flies expressing a *G9a* transgene in (**A**) hemocytes or (**B**) glial cells upon DCV infection (1,000 TCID_50_ units). The transcription factor Gal4 is expressed under control of (**A**) the hemocyte-specific *Hemolectin* promoter (*Hml*-Gal4), or (**B**) the glial cell-specific *repo* promoter (*repo-Gal4*), and binds to the Upstream Activating Sequences to induce expression of the *G9a* transgene (*UAS-G9a*). Control flies expressing only the *repo-Gal4* or the *UAS-G9a* transgenes were included as controls. A representative experiment of five (**A**) and two (**B**) independent experiments with 20 males flies for each genotype is shown.
(TIF)

S3 Fig *G9a* mutants have a functional RNAi response. (**A**) Eye phenotype of wild-type or *G9a* mutant flies (3 to 5-day-old) expressing an RNAi-inducing inverted repeat RNA targeting the Drosophila Inhibitor of Apoptosis *thread* (*th* ^RNAi^). As controls, eyes of wild-type and *G9a* mutant flies not expressing the inverted repeat are shown. Five representative images are shown for each genotype. (**B**) *In vivo* RNAi reporter assay in adult flies. Fluc and RLuc reporter plasmids were transfected along with FLuc specific dsRNA or non-specific control dsRNA in *G9a* ^-/-^ and *AGO2* ^-/-^ mutant flies and their wild-type controls (*G9a* ^+/+^ and *w* ^1118^, respectively). Reporter gene activity was measured at three days after transfection and fold silencing by Fluc dsRNA relative to control GFP dsRNA was calculated. Results are expressed as percentage of silencing relative to wild-type flies (*w* ^1118^ and *G9a* ^+/+^). Bars represent means and s.d. of three pools of five flies for each genotype. Data are from one experiment representative of two (**A**) and three (**B**) independent experiments.
(TIF)

S4 Fig DCV-induced transcriptome in *G9a* mutants. (**A,B**) List of genes that are expressed ≥2-fold upon DCV infection (relative to mock) in both wild-type and *G9a* mutant flies in (**A**) whole flies or (**B**) fat bodies. (**C,D**) Gene ontology (GO) analysis of genes that are expressed at ≥2-fold higher levels in DCV infected *G9a* mutants than in infected wild-type flies. All significantly enriched GO terms of level 3 are shown (*P* < 0.05 in a hypergeometric test with Benjamini & Hochberg correction), with their respective fold enrichment (defined as the ratio of the frequency in the dataset to the genome-wide frequency). Data are from whole flies (**C**) or dissected fat bodies (**D**).
(TIF)

S5 Fig Primer location and specificity controls for ChIP-qPCR. (**A-D**) Schematic representation of *G9a* target loci within the *Socs36E* (**A**), *TotM* (**B**), *vir-1* (**C**) and *dPIAS* (**D**) genes, defined as genomic regions in which the H3K9me2 mark is present in wild-type flies but not in *G9a* mutants, in a previous study [20]. The arrow represents the position of the amplicon generated by qPCR after Chromatin-Immunoprecipitation (ChIP-qPCR). Blue and red plots represent H3K9me2 levels in wild-type and *G9a* mutants, respectively. (**E-J**) ChIP-qPCR in the *moca* (**E**,**G**,**I**) and *domeless* (**F**,**H**,**J**) loci, performed on fat bodies of wild-type or *G9a* mutant flies with aspecific anti-IgG control (**E**,**F**), anti-H3 (**G**,**H**), and anti-H3K9me2 (**I**,**J**) antibodies. Data are presented as percentage of input, calculated by dividing the signal obtained after IP by the signal obtained from the input. The results indicate that there is very low aspecific binding of chromatin to the control IgG antibody (**E**,**F**), and that H3 levels are similar on the *moca* and *domeless* loci of wild-type flies and *G9a* mutants (**G**,**H**). Moreover, these results show that the *moca* locus is depleted of H3K9me2 marks both in wild-type and *G9a* mutant flies (**I**), and that the *domeless* locus is depleted of H3K9me2 in *G9a* mutants (**J**). Data are means and s.d. of three independent pools of 80 female fat bodies for each genotype. **P < 0.01 (Student’s t-test).
(TIF)

S6 Fig Genetic interaction between *G9a* and the Jak-Stat pathway. (**A**,**B**) Survival upon mock infection of wild-type or *G9a* mutant and wild-type mutant flies overexpressing (**A**) dome^ΔCyt^, or (**B**) *Socs36E*. These mock infections were run in parallel to the experiments of Fig 6A and 6B. (**C,D**) Survival upon DCV infection (100 TCID_50_ units) of wild-type or *G9a* mutant flies overexpressing (**C**) dome^ΔCyt^, or (**D**) *Socs36E*, as described in Fig 6. Control flies expressing only the *Act-Gal4*, the *UAS-dome* ^ΔCyt^, or the *UAS-Socs36E* transgenes and mock infections were included as controls (see S11 Dataset). Data are means and s.d. of three independent pools of at least 15 male flies for each genotype.
(TIF)

S7 Fig Enrichment of immune-related GO terms and predicted promoter binding sites in *G9a*- and *GLP*-depleted mice. We analyzed microarray data published by Schaefer *et al*. [18] for enrichment of GO terms and transcription factor binding sites among genes with ≥2-fold expression in the hippocampus of mice depleted of (**A**) *G9a*, or its paralog (**B**) *GLP* in post-natal forebrain neurons. Significantly enriched GO categories are shown (*P* < 0.05 in a hypergeometric test with Benjamini & Hochberg correction). Pscan was used to predict transcription factor binding sites in the 500-bp region upstream of the transcription start site using the TRANSFAC database. The top-10 significantly enriched transcription factors compared to the genome-wide mean are shown (*P* < 0.05 in a z-test).
(TIF)

S1 Dataset Data related to Fig 1. (XLSX)

S2 Dataset Data related to Fig 2. (XLSX)

S3 Dataset Data related to Fig 3. (XLSX)

S4 Dataset Data related to Fig 5. (XLSX)

S5 Dataset Data related to Fig 6. (XLSX)

S6 Dataset Data related to Fig 7. (XLSX)

S7 Dataset Data related to S1 Fig. (XLSX)

S8 Dataset Data related to S2 Fig. (XLSX)

S9 Dataset Data related to S3 Fig. (XLSX)

S10 Dataset Data related to S5 Fig. (XLSX)

S11 Dataset Data related to S6 Fig. (XLSX)
