# Supplementary figures and images for: The Epigenetic Regulator G9a Mediates Tolerance to RNA Virus Infection in Drosophila
Source: PLoS Pathog. 2015 Apr 16;11(4):e1004692. doi: 10.1371/journal.ppat.1004692 (PMC4399909; doi:10.1371/journal.ppat.1004692)

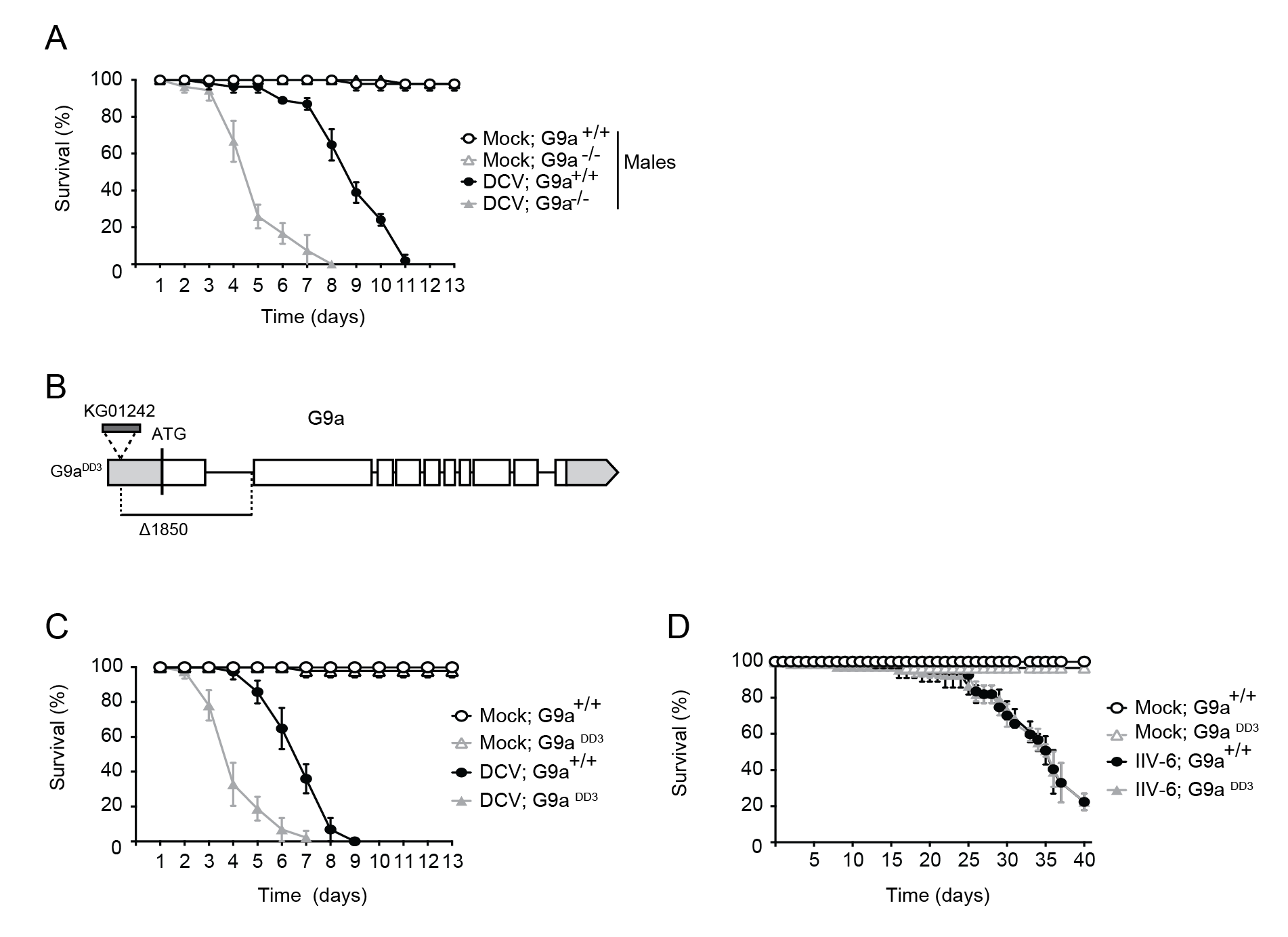

Supplement: S1 Fig — (A) Survival of male, wild-type or G9a mutant flies upon DCV infection, or Tris buffer control (mock). The mean survival is 8.9 days for wild-type flies, and 5.1 days for G9a mutants (P < 0.001). (B) Structure of the G9a locus. Boxes represent exons (5’ and 3’-untranslated regions in gray, and coding sequence in white). The KG01242 P-element insertion site that was used to generate the G9a DD3 allele is depicted by dashed lines. Size and location of the G9a deletion in the G9a DD3 allele are indicated. (C,D) Survival of wild-type or G9a DD3 mutants infected with (C) DCV, (D) IIV-6, or with Tris buffer as a control (mock). Upon DCV infection (C), the mean survival is 6.9 days for wild-type flies, and 4.4 days for G9a DD3 mutants (P < 0.001). Data represent means and s.d. of three biological replicates of 15 male flies (A) or 20 female flies (C,D) per replicate for each genotype. A representative experiment of 3 independent experiments is shown. (TIF) [file ppat.1004692.s001.tif]

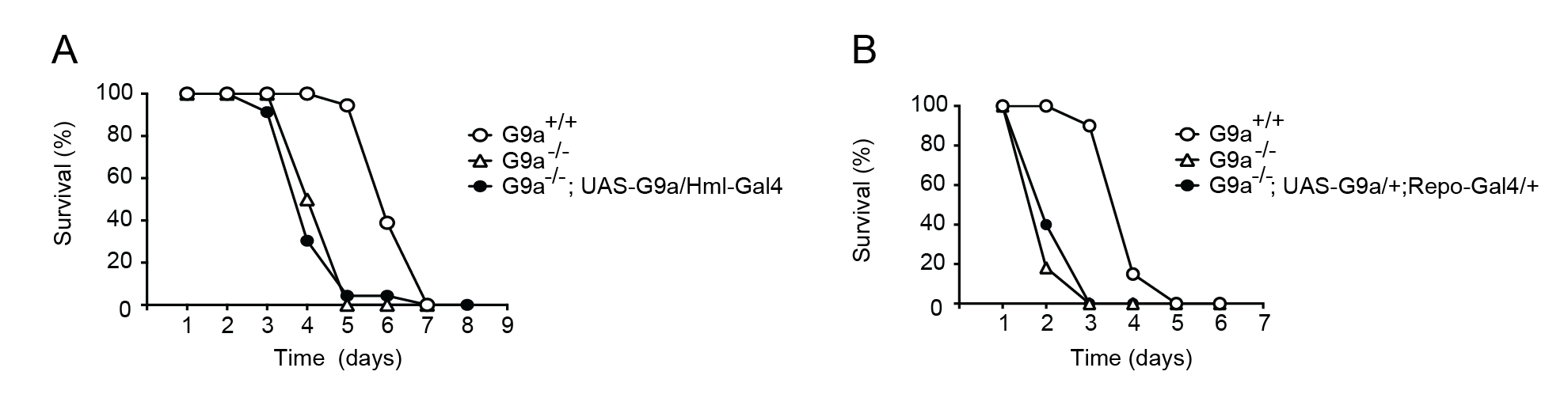

Supplement: S2 Fig — (A,B) Survival of wild-type or G9a -/- flies expressing a G9a transgene in (A) hemocytes or (B) glial cells upon DCV infection (1,000 TCID50 units). The transcription factor Gal4 is expressed under control of (A) the hemocyte-specific Hemolectin promoter (Hml-Gal4), or (B) the glial cell-specific repo promoter (repo-Gal4), and binds to the Upstream Activating Sequences to induce expression of the G9a transgene (UAS-G9a). Control flies expressing only the repo-Gal4 or the UAS-G9a transgenes were included as controls. A representative experiment of five (A) and two (B) independent experiments with 20 males flies for each genotype is shown. (TIF) [file ppat.1004692.s002.tif]

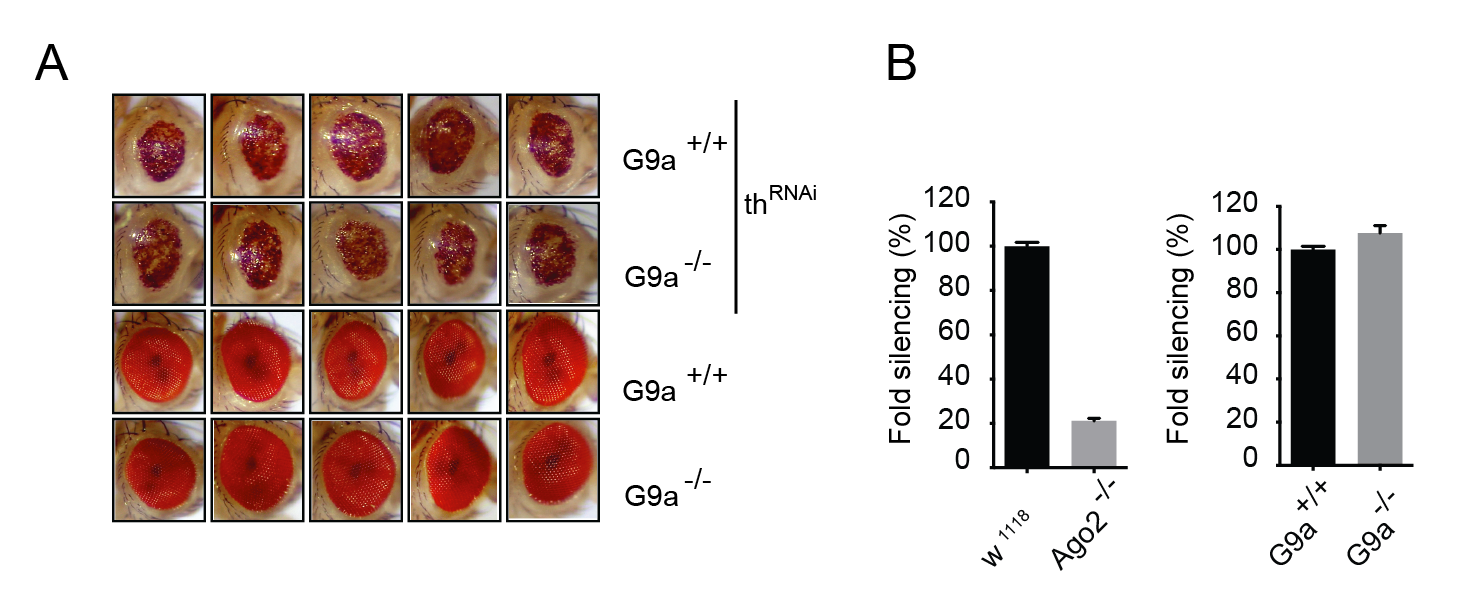

Supplement: S3 Fig — (A) Eye phenotype of wild-type or G9a mutant flies (3 to 5-day-old) expressing an RNAi-inducing inverted repeat RNA targeting the Drosophila Inhibitor of Apoptosis thread (th RNAi). As controls, eyes of wild-type and G9a mutant flies not expressing the inverted repeat are shown. Five representative images are shown for each genotype. (B) In vivo RNAi reporter assay in adult flies. Fluc and RLuc reporter plasmids were transfected along with FLuc specific dsRNA or non-specific control dsRNA in G9a -/- and AGO2 -/- mutant flies and their wild-type controls (G9a +/+ and w 1118, respectively). Reporter gene activity was measured at three days after transfection and fold silencing by Fluc dsRNA relative to control GFP dsRNA was calculated. Results are expressed as percentage of silencing relative to wild-type flies (w 1118 and G9a +/+). Bars represent means and s.d. of three pools of five flies for each genotype. Data are from one experiment representative of two (A) and three (B) independent experiments. (TIF) [file ppat.1004692.s003.tif]

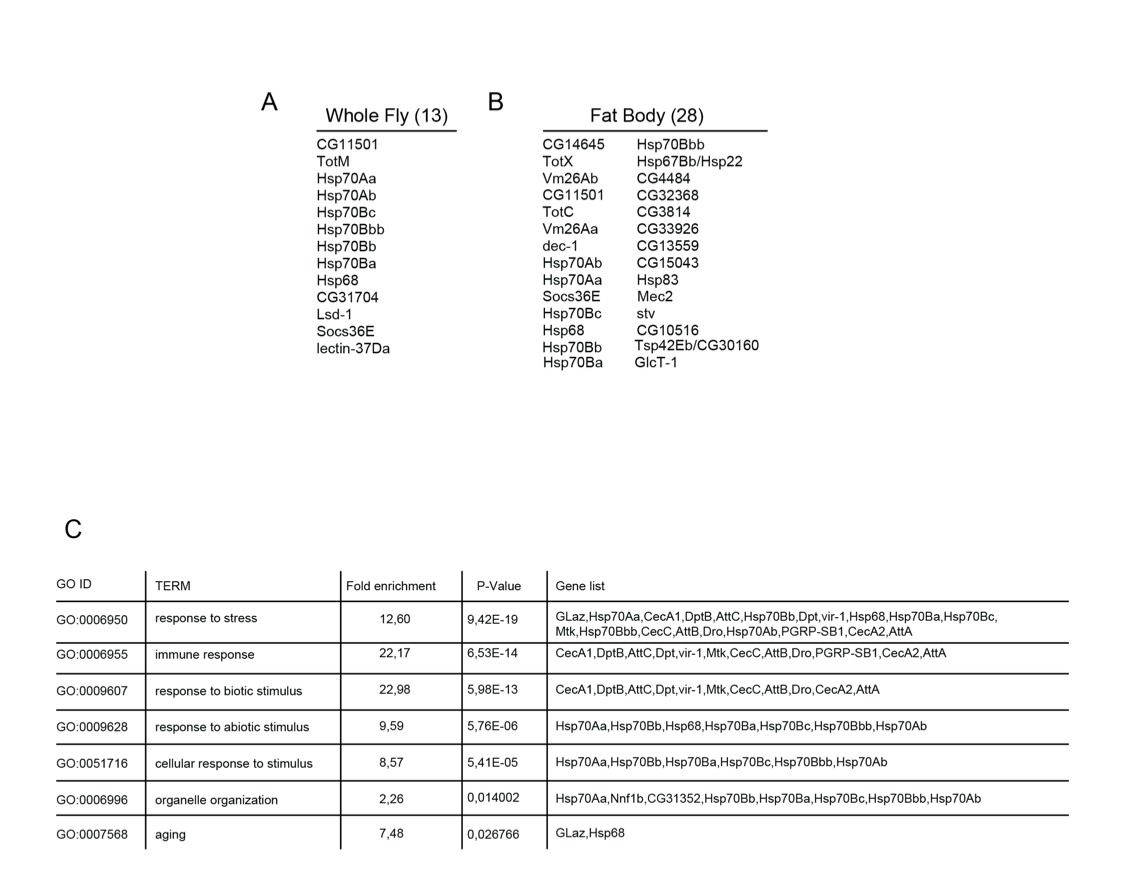

Supplement: S4 Fig — (A,B) List of genes that are expressed ≥2-fold upon DCV infection (relative to mock) in both wild-type and G9a mutant flies in (A) whole flies or (B) fat bodies. (C,D) Gene ontology (GO) analysis of genes that are expressed at ≥2-fold higher levels in DCV infected G9a mutants than in infected wild-type flies. All significantly enriched GO terms of level 3 are shown (P < 0.05 in a hypergeometric test with Benjamini & Hochberg correction), with their respective fold enrichment (defined as the ratio of the frequency in the dataset to the genome-wide frequency). Data are from whole flies (C) or dissected fat bodies (D). (TIF) [file ppat.1004692.s004.tif]

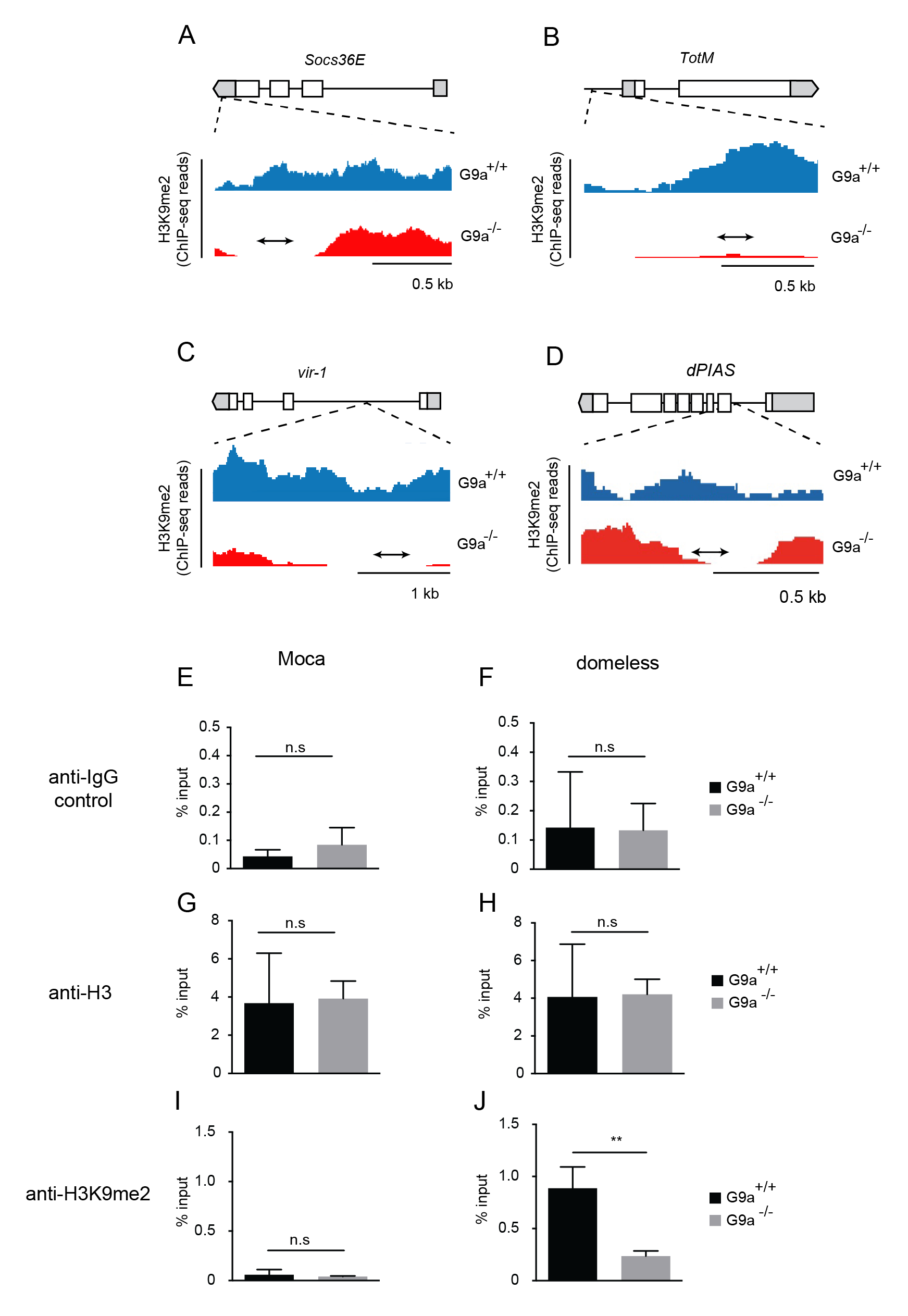

Supplement: S5 Fig — (A-D) Schematic representation of G9a target loci within the Socs36E (A), TotM (B), vir-1 (C) and dPIAS (D) genes, defined as genomic regions in which the H3K9me2 mark is present in wild-type flies but not in G9a mutants, in a previous study [20]. The arrow represents the position of the amplicon generated by qPCR after Chromatin-Immunoprecipitation (ChIP-qPCR). Blue and red plots represent H3K9me2 levels in wild-type and G9a mutants, respectively. (E-J) ChIP-qPCR in the moca (E,G,I) and domeless (F,H,J) loci, performed on fat bodies of wild-type or G9a mutant flies with aspecific anti-IgG control (E,F), anti-H3 (G,H), and anti-H3K9me2 (I,J) antibodies. Data are presented as percentage of input, calculated by dividing the signal obtained after IP by the signal obtained from the input. The results indicate that there is very low aspecific binding of chromatin to the control IgG antibody (E,F), and that H3 levels are similar on the moca and domeless loci of wild-type flies and G9a mutants (G,H). Moreover, these results show that the moca locus is depleted of H3K9me2 marks both in wild-type and G9a mutant flies (I), and that the domeless locus is depleted of H3K9me2 in G9a mutants (J). Data are means and s.d. of three independent pools of 80 female fat bodies for each genotype. **P < 0.01 (Student’s t-test). (TIF) [file ppat.1004692.s005.tif]

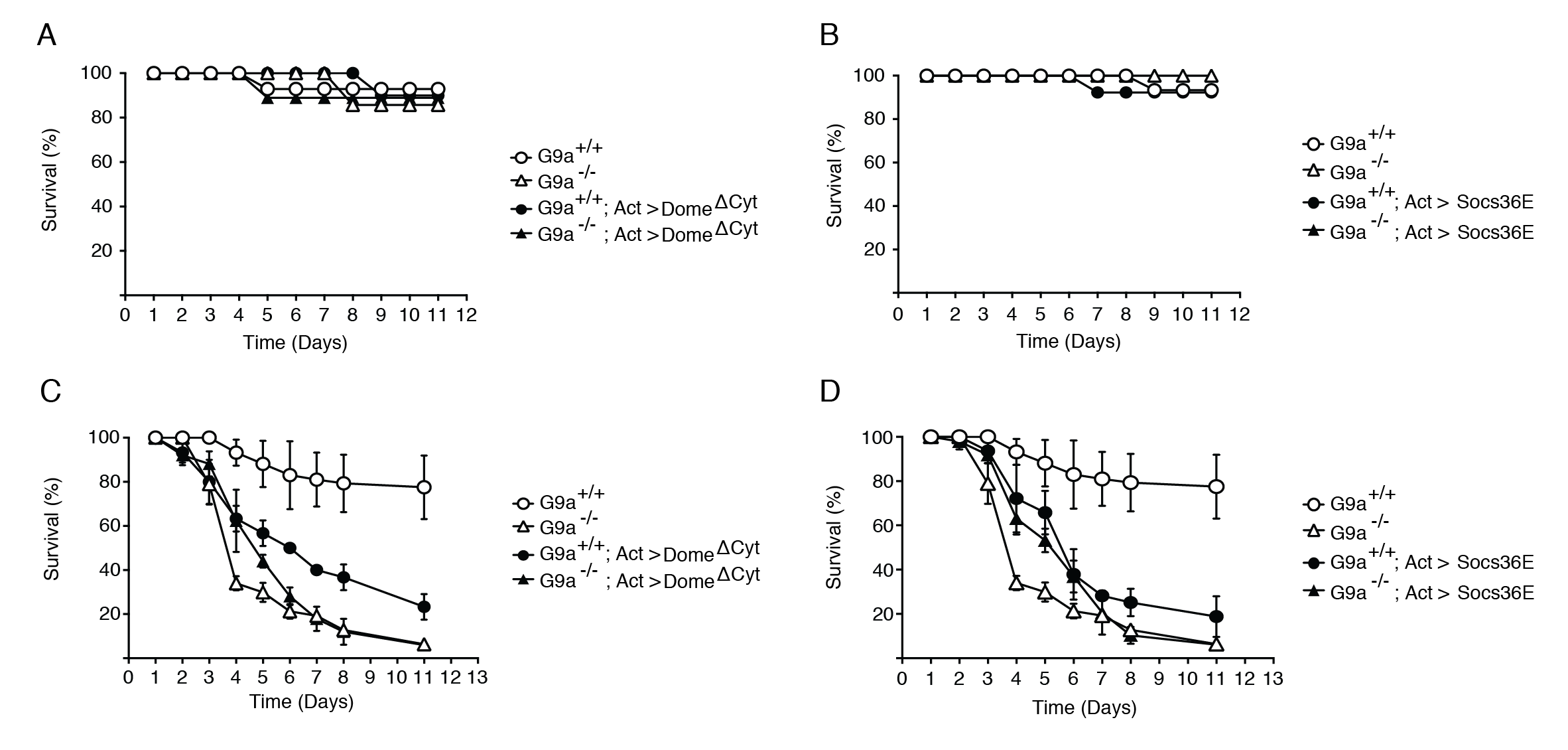

Supplement: S6 Fig — (A,B) Survival upon mock infection of wild-type or G9a mutant and wild-type mutant flies overexpressing (A) domeΔCyt, or (B) Socs36E. These mock infections were run in parallel to the experiments of Fig 6A and 6B. (C,D) Survival upon DCV infection (100 TCID50 units) of wild-type or G9a mutant flies overexpressing (C) domeΔCyt, or (D) Socs36E, as described in Fig 6. Control flies expressing only the Act-Gal4, the UAS-dome ΔCyt, or the UAS-Socs36E transgenes and mock infections were included as controls (see S11 Dataset). Data are means and s.d. of three independent pools of at least 15 male flies for each genotype. (TIF) [file ppat.1004692.s006.tif]

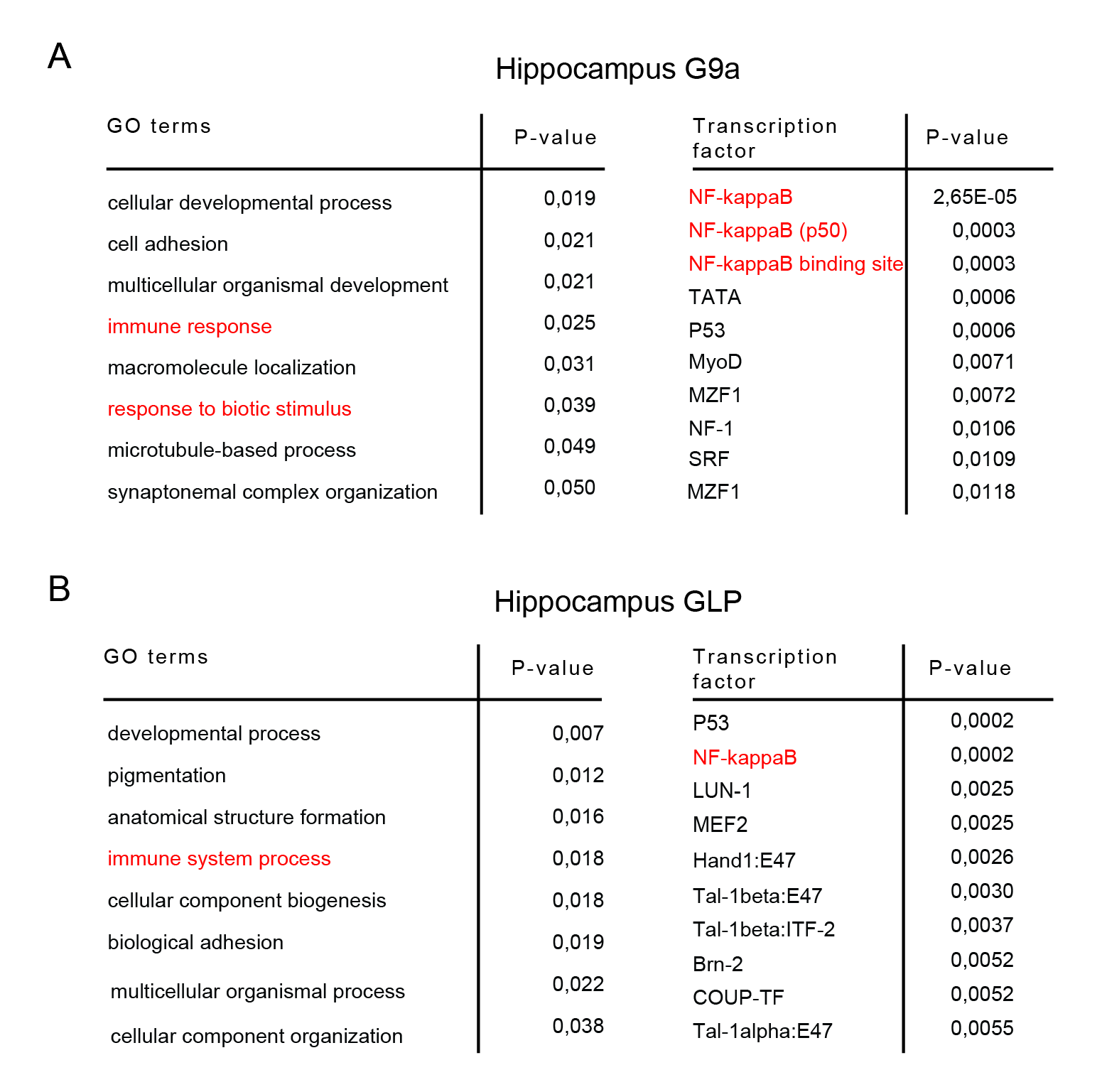

Supplement: S7 Fig — We analyzed microarray data published by Schaefer et al. [18] for enrichment of GO terms and transcription factor binding sites among genes with ≥2-fold expression in the hippocampus of mice depleted of (A) G9a, or its paralog (B) GLP in post-natal forebrain neurons. Significantly enriched GO categories are shown (P < 0.05 in a hypergeometric test with Benjamini & Hochberg correction). Pscan was used to predict transcription factor binding sites in the 500-bp region upstream of the transcription start site using the TRANSFAC database. The top-10 significantly enriched transcription factors compared to the genome-wide mean are shown (P < 0.05 in a z-test). (TIF) [file ppat.1004692.s007.tif]
